# Essential functions of mosquito ecdysone importers in development and reproduction

**DOI:** 10.1073/pnas.2202932119

**Published:** 2022-06-13

**Authors:** Lewis V. Hun, Naoki Okamoto, Eisuke Imura, Roilea Maxson, Riyan Bittar, Naoki Yamanaka

**Affiliations:** ^a^Department of Entomology, Institute for Integrative Genome Biology, University of California, Riverside, CA 92521;; ^b^Life Science Center for Survival Dynamics, Tsukuba Advanced Research Alliance, University of Tsukuba, Tsukuba, Ibaraki 305-8577, Japan

**Keywords:** ecdysone, organic anion-transporting polypeptide (OATP), *Aedes aegypti*, *Drosophila melanogaster*, vitellogenesis

## Abstract

The primary insect steroid hormone ecdysone requires a membrane transporter to enter its target cells. Although an organic anion-transporting polypeptide (OATP) named Ecdysone Importer (EcI) serves this role in the fruit fly *Drosophila melanogaster* and most likely in other arthropod species, this highly conserved transporter is apparently missing in mosquitoes. Here we report three additional OATPs that facilitate cellular incorporation of ecdysone in *Drosophila* and the yellow fever mosquito *Aedes aegypti*. These additional ecdysone importers (EcI-2, -3, and -4) are dispensable for development and reproduction in *Drosophila*, consistent with the predominant role of EcI. In contrast, in *Aedes*, EcI-2 is indispensable for ecdysone-mediated development, whereas EcI-4 is critical for vitellogenesis induced by ecdysone in adult females. Altogether, our results indicate unique and essential functions of these additional ecdysone importers in mosquito development and reproduction, making them attractive molecular targets for species- and stage-specific control of ecdysone signaling in mosquitoes.

## Significance

Steroid hormones control sexual maturation and reproduction in insects and humans alike. The insect steroid hormone ecdysone uses a membrane transporter named Ecdysone Importer (EcI) to enter cells and promote these physiological processes, but *EcI* is unexpectedly missing in mosquito genomes. Using the yellow fever mosquito *Aedes aegypti*, here we show that mosquitoes use alternative ecdysone importers to facilitate ecdysone-dependent development and reproduction. These transporters are also present in other insects, including fruit flies, but they are dispensable for fly development and reproduction likely due to their limited expression patterns. Our results thus indicate that differential expression of steroid hormone importers enables tissue- and stage-specific hormone responses, and some importers can obtain critical physiological functions only in certain species.

Ecdysone and other ecdysteroids are a group of steroid hormones that control various aspects of insect development and reproduction (1). Once released into the hemolymph, ecdysone (more specifically, its active form 20-hydroxyecdysone or 20E and related ecdysteroids) enters its target cells to bind to a nuclear receptor named the ecdysone receptor (EcR), which forms a heterodimer with another nuclear receptor Ultraspiracle and induces gene expression (2–5). Although it has long been assumed that ecdysone can pass through the cell membrane through simple diffusion, we recently demonstrated that an organic anion-transporting polypeptide (OATP), which we named Ecdysone Importer (EcI), is required for cellular uptake of ecdysone in the fruit fly *Drosophila melanogaster* (6). *EcI* orthologs are found among a wide variety of insects (6), and it is likely that its critical function as a mediator of ecdysone signaling is highly conserved in other insect species (7).

Mosquitoes are the deadliest disease vectors for humans. The yellow fever mosquito *Aedes aegypti* is the primary vector for arboviruses, including Zika, yellow fever, chikungunya, and dengue viruses, which are of global health concern due to their rapid increases in the geographical distribution (8, 9). Critical functions of ecdysone signaling in *Aedes* development and reproduction are extensively investigated (10–13), making it an important molecular target for control agents against this deadly virus vector. Surprisingly, however, even in well-annotated mosquito genomes (14–19), an *EcI* ortholog cannot be found, suggesting the existence of an additional membrane transporter(s) for ecdysone.

In this study, we identified additional OATPs that facilitate cellular uptake of ecdysone in *Drosophila* and *Aedes*. These additional ecdysone importer-encoding genes, *EcI-2*, -*3*, and -*4*, are dispensable for normal development and reproduction in *Drosophila*, confirming the predominant role of *EcI* in flies. In contrast, CRISPR/Cas9-mediated mutagenesis and RNA interference (RNAi)-mediated knockdown experiments in *Aedes* suggest that *EcI-2* is critical for ecdysone-mediated developmental progression, while *EcI-4* is most important for vitellogenesis induced by ecdysone in adult females. Collectively, our results indicate unique functions of these additional ecdysone importers in mosquitoes, making them attractive targets for species- and stage-specific control of mosquito ecdysone signaling.

## Results

### Identification of Additional Ecdysone Importers in *Drosophila* and *Aedes*.

**Fig. 1.**
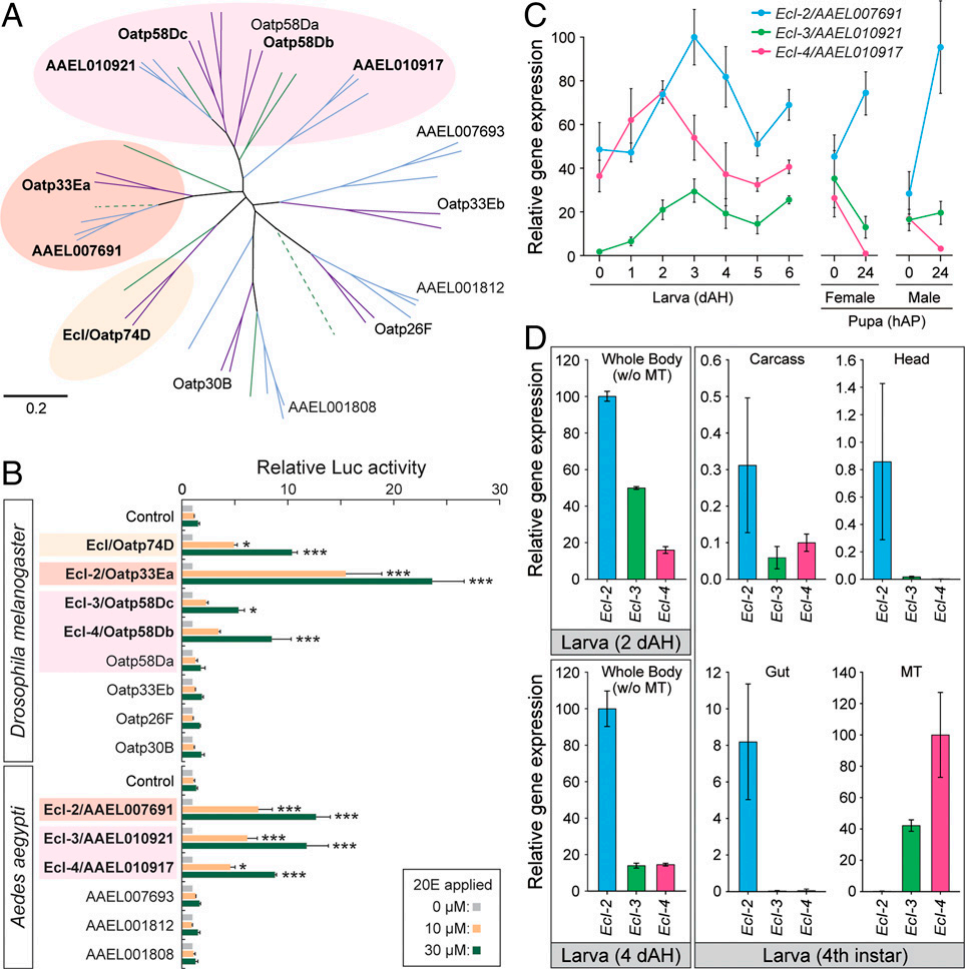
Identification of additional ecdysone importers in *Drosophila* and *Aedes*. (*A*) Neighbor-joining unrooted phylogenetic tree of full-length OATP proteins from representative dipteran insect species. Purple, flies (*D. melanogaster* and *M. domestica*); green, sand flies (*P. papatasi*); blue, mosquitoes (*Ae. aegypti*, *A. gambiae*, and *Culex quinquefasciatus*). Dotted lines indicate pseudogenes. *Drosophila* and *Aedes* OATPs are labeled. Protein names and GenBank accession numbers are listed in *SI Appendix*, Table S1. Scale bar indicates an evolutionary distance of 0.2 amino acid substitutions per position. (*B*) Luciferase (Luc) reporter activities in response to 10 μM or 30 μM 20E in HEK293 cells expressing *Drosophila* or *Aedes* OATPs. The cells were transfected with modified *EcR* (*VgEcR*) and *RXR*, along with each OATP-containing vector and luciferase reporter plasmids. Values are relative to the basal level (0 M 20E). All values are the means ± SEM (*n* = 2 to 4). *P < 0.05, ***P < 0.001 from one-way ANOVA followed by Dunnett’s multiple comparison test as compared to the response of the control cells to the same concentration of 20E. (*C*) Relative expression levels of ecdysone importer genes in the whole body during *Aedes* development, as assessed by qRT-PCR. Values are shown as percentages relative to the highest expression level of *EcI-2*. dAH, days after hatching, hAP, hours after pupation. All values are the means ± SEM (*n* = 3). (*D*) Tissue-specific expression of ecdysone importer genes during *Aedes* development, as assessed by qRT-PCR. Whole body samples without Malpighian tubules from early developmental stages (2 and 4 d after hatching) or individual tissues of fourth instar larvae were collected. Values are shown as percentages relative to the expression levels of *EcI-2* in early developmental stages, or *EcI-4* in the Malpighian tubule in the fourth instar. MT, Malpighian tubule. All values are the means ± SEM (*n* = 3).

In order to identify all OATPs in mosquitoes and related dipteran species, we thoroughly searched for orthologs of *Drosophila* OATPs in flies (*Musca domestica*), sand flies (*Phlebotomus papatasi*), and mosquitoes (*Ae. aegypti*, *Anopheles gambiae*, and *Culex quinquefasciatus*). Our comprehensive BLAST search identified eight OATPs in *M. domestica*, eight OATPs in *P. papatasi*, six OATPs in *Ae. aegypti*, six OATPs in *An. gambiae*, and seven OATPs in *C. quinquefasciatus* (*SI Appendix*, Table S1). Phylogenetic analysis of these dipteran OATPs confirmed absence of *EcI* orthologs in mosquitoes (Fig. 1*A*).

To identify additional ecdysone importers, we used an ecdysteroid-inducible gene expression system in HEK293 cells (20–22) to test whether the other OATPs in *Drosophila* and *Aedes* can facilitate ecdysone signaling in a heterologous system. This led to identification of three additional ecdysone importers in each species: Oatp33Ea, Oatp58Dc, and Oatp58Db in *Drosophila*, and AAEL007691, AAEL010921, and AAEL010917 in *Aedes* (Fig. 1*B*). Based on phylogenetic similarities, these additional ecdysone importers in each species were named EcI-2, -3, and -4, respectively. Each of these OATPs shows ∼60 to 74% amino acid sequence similarities between *Drosophila* and *Aedes*.

Temporal expression of *EcI-2*, -*3*, and -*4* fluctuates during development in mosquitoes and flies (Fig. 1*C* and *SI Appendix*, Fig. S1*A*). With regard to tissue-specific expression, we previously showed that *EcI-2* is highly expressed in the gut, whereas *EcI-3* and *EcI-4* are predominantly expressed in the Malpighian tubules in *Drosophila* larvae (6). Consistent with this, we observed high expression of *EcI-2* in the gut and *EcI-3* and *EcI-4* in the Malpighian tubules, respectively, in *Aedes* fourth instar larvae (Fig. 1*D*). Importantly, in addition to its strong expression in the gut, *Aedes EcI-2* is expressed at higher levels in the head and carcass as compared to the other ecdysone importers in fourth instar larvae. Predominant expression of *EcI-2* outside of the Malpighian tubules was further confirmed in earlier larval stages (Fig. 1*D*), suggesting its potential ubiquitous function during larval development.

To confirm their ecdysone importer activities in vivo, *Drosophila EcI-2*, -*3*, and -*4* were ubiquitously expressed in *EcI* null flies using the Gal4/UAS system and tested whether they can rescue the first instar arrest phenotype caused by loss of ecdysone signaling (6). As shown in *SI Appendix*, Fig. S1*B*, all of these additional ecdysone importers significantly rescued the first instar arrest phenotype of *EcI* null flies, although the rescued animals could only develop into the second instar as compared to further rescue achieved by expression of *UAS-EcI*. Importantly, the degree of partial rescue observed by ectopic expression was similar among the three additional importers (*SI Appendix*, Fig. S1*B*), suggesting that their functions as ecdysone importers are comparable in vivo.

### Unique Requirement of Additional Ecdysone Importers during Mosquito Embryogenesis.

**Fig. 2.**
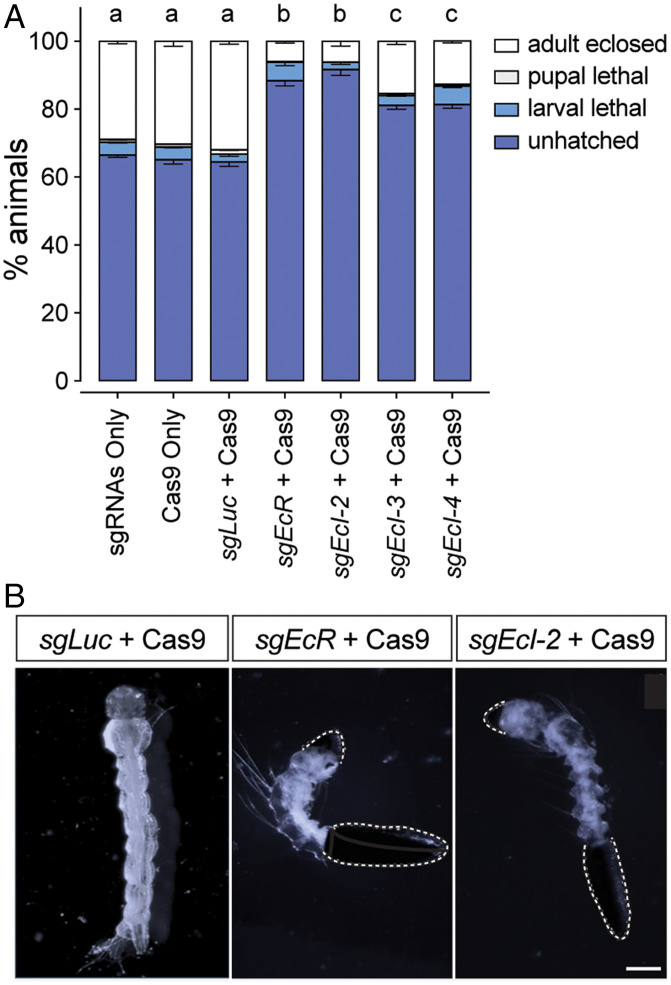
*Aedes* ecdysone importers are required for embryogenesis. (*A*) Lethal stages of *Aedes* embryos injected with Cas9 protein and/or sgRNAs against control (*sgLuc*), *EcR* (*sgEcR*), or ecdysone importer genes (*sgEcI-2*, *sgEcI-3*, and *sgEcI-4*). Total 450 eggs were injected in three independent batches for each treatment, and their lethal stages were monitored thereafter. All values are the means ± SEM. Same letters on *Top* of the bars indicate statistically insignificant differences in embryonic lethality based on one-way ANOVA with Tukey's honestly significant difference (HSD) test. (*B*) Hatching defects observed in *EcR* and *EcI-2* mutants. As compared to control (*sgLuc* + Cas9) that hatched normally, *EcR* or *EcI-2* mutagenesis caused hatching defects in some larvae, where the eggshell remained attached (dotted white lines). (Scale bar, 0.5 mm.)

We next conducted CRISPR/Cas9-mediated mutagenesis of *EcI-2*, -*3*, and -*4* in *Drosophila* and *Aedes*, aiming to investigate their functions during the life cycle. In *Drosophila*, neither individual mutants nor combination mutants showed any discernible developmental or reproductive defects (*SI Appendix*, Figs. S2 and S3 and Tables S2 and S3), consistent with the role of *EcI* as the predominant ecdysone importer in flies (6). In contrast, when single guide RNA (sgRNA) for each gene (*SI Appendix*, Fig. S4) was injected with Cas9 protein into *Aedes* eggs, all ecdysone importer mutants showed significant embryonic lethality as compared to controls (Fig. 2*A*). Importantly, only *EcI-2* mutants showed levels of embryonic lethality comparable to *EcR* knockout animals (Fig. 2*A*). Some of the *EcI-2* mutants also showed hatching defects similar to *EcR* mutants (Fig. 2*B*), suggesting a critical function of EcI-2 in ecdysone signaling during mosquito embryogenesis.

### *EcI-2* Is Required for Mosquito Larval Development.

**Fig. 3.**
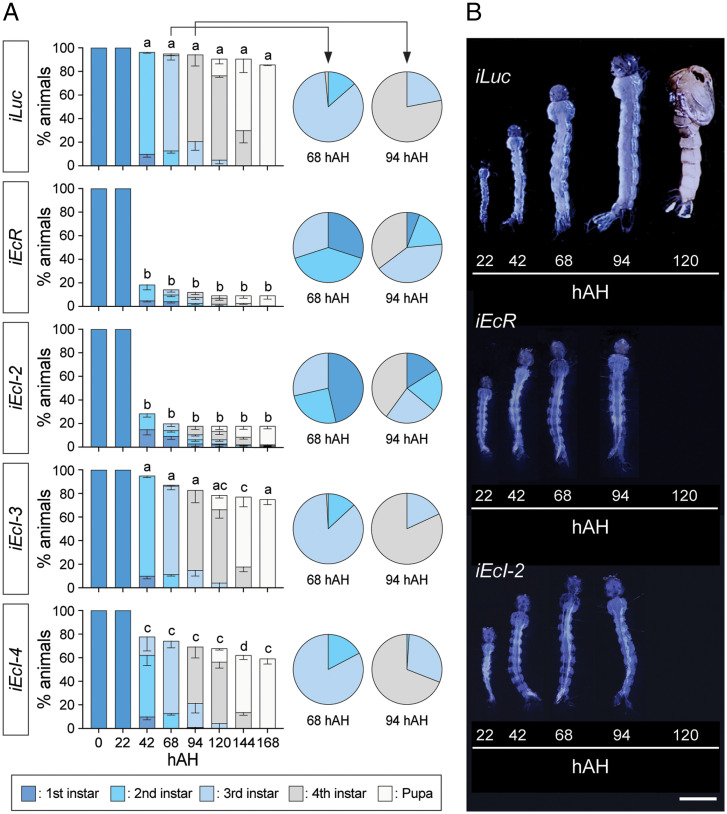
*Aedes EcI-2* is required for larval developmental transitions. (*A*) Developmental progression and survival rate (%) of *Luc* RNAi (*iLuc*; control), *EcR* RNAi (*iEcR*), and *EcI-2*, -*3*, and -*4* RNAi (*iEcI-2*, *iEcI-3*, and *iEcI-4*) animals. Color bars indicate developmental stages determined by stage-specific morphologic features such as the head capsule size and siphon length (42, 43). All values are the means ± SEM from seven independent experiments with 20 individuals in each replicate. hAH, hours after hatching. Between 42 and 168 hAH, same letters on *Top* of the bars indicate statistically insignificant differences in survival rate based on one-way ANOVA followed by Bonferroni’s multiple comparison test among RNAi animals at the same time point. Developmental stages of surviving larvae at 68 and 94 hAH are shown on the *Right* as pie charts. (*B*) Developmental progression of animals treated with dsRNA targeting *Luc* (*iLuc*; control), *EcR* (*iEcR*), or *EcI-2* (*iEcI-2*). Representative images of animals at various time points were combined into single panels. (Scale bar, 1 mm.)

In order to circumvent embryonic lethality in ecdysone importer mutants, we undertook an RNAi approach to further investigate their potential involvement in ecdysone signaling at later developmental stages. Newly hatched *Aedes* larvae were soaked in double-strand RNA (dsRNA) solutions to suppress expression of each ecdysone importer or *EcR*, which resulted in ∼50 to 60% reduction of mRNA levels for each target gene (*SI Appendix*, Fig. S5). Periodic monitoring of their developmental stages and lethality revealed that *EcR* knockdown induced high (80 to 90%) lethality beginning 42 h after hatching (hAH), consistent with the critical function of ecdysone signaling in molting induction during larval stages (Fig. 3*A*). Likewise, knockdown of *EcI-2*, but not *EcI-3* or *EcI-4*, induced high lethality statistically indistinguishable from *EcR* knockdown during larval development. Importantly, after 68 hAH, many surviving *EcR* RNAi and *EcI-2* RNAi larvae remained as younger instars compared to control animals (Fig. 3*A*), and their size increase was clearly inhibited (Fig. 3*B*). This developmental arrest phenotype in *EcR* RNAi and *EcI-2* RNAi animals was further confirmed by detailed measurement of head size and siphon length at two different time points during larval development (*SI Appendix*, Fig. S6). Furthermore, expression of an ecdysone-inducible gene, *E74B*, was suppressed by ∼70% in *EcI-2* RNAi animals, as compared to milder (∼40%) reduction in *EcI-3* or *EcI-4* RNAi animals (*SI Appendix*, Fig. S7). This strong inhibition of *E74B* expression in *EcI-2* RNAi animals was comparable to its suppression induced by RNAi knockdown of *EcR* (72%) or *shade* (74%), the gene encoding a cytochrome P450 enzyme required for 20E production (23, 24). Overall, these results further suggest involvement of EcI-2 in ecdysone signaling during mosquito development.

**Fig. 4.**
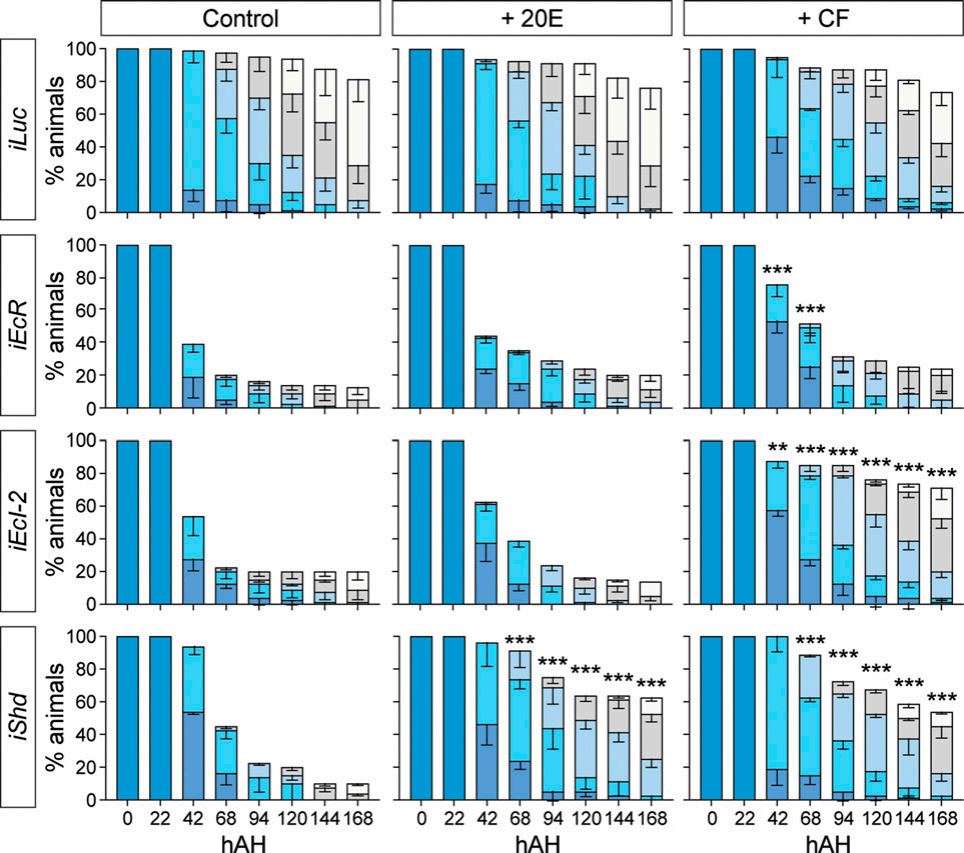
A nonsteroidal ecdysone agonist can rescue developmental arrest caused by *EcI-2* knockdown in *Aedes*. Developmental progression and survival rate (%) of *Luc* RNAi (*iLuc*; control), *EcR* RNAi (*iEcR*), *EcI-2* RNAi (*iEcI-2*), and *shade* RNAi (*iShd*) animals treated with 20E or CF. Color bars indicate developmental stages as shown in Fig. 3*A*. All values are the means ± SEM from four independent experiments with 20 individuals in each replicate. **P < 0.01, ***P < 0.001 from one-way ANOVA followed by Bonferroni’s multiple comparison test as compared to the survival rate of control (no agonist treatment) of the same RNAi animal at the same time point.

We next attempted to rescue the larval arrest phenotype by administration of two different EcR agonists: the endogenous ecdysteroid 20E and a nonsteroidal insecticide chromafenozide (CF) (Fig. 4). As we have previously shown that CF enters cells independently of EcI (6, 25, 26), we reasoned that CF can activate EcR even in the absence of ecdysone importers, thereby rescuing the developmental defect caused by *EcI-2* knockdown. Indeed, CF but not 20E significantly rescued the larval arrest phenotype in *EcI-2* RNAi animals, whereas these agonists only partially rescued the larval lethality, if at all, caused by *EcR* knockdown (Fig. 4). Importantly, both CF and 20E significantly rescued developmental arrest caused by knockdown of *shade*, confirming the potency of these compounds as EcR agonists in vivo. Collectively, these results indicate that EcR remains functional in the absence of EcI-2, and that EcI-2 is required for 20E to access EcR and induce larval development in mosquitoes.

### *EcI-4* Is Required for Mosquito Vitellogenesis.

In adult female mosquitoes, vitellogenesis after the blood meal is primarily controlled by 20E. 20E up-regulates expression of ecdysone-inducible genes, whose products in turn promote expression of yolk protein precursors such as vitellogenin (Vg) in the fat body (10, 11). When expression levels of ecdysone importers were periodically monitored after the blood meal, we observed significant fluctuation of their expression in all tissues observed, potentially reflecting dynamic changes in ecdysone signaling after blood feeding (*SI Appendix*, Fig. S8). Importantly, in the abdomen where the fat body is located, expression of *EcI-4* was significantly up-regulated as compared to the other ecdysone importer genes between 16 and 20 h after the blood meal (*SI Appendix*, Fig. S8*A*). This precedes the peak of ecdysone levels observed ∼18 to 24 h after blood feeding in adult females (27), indicating a critical function of EcI-4 in ecdysone-induced vitellogenesis.

**Fig. 5.**
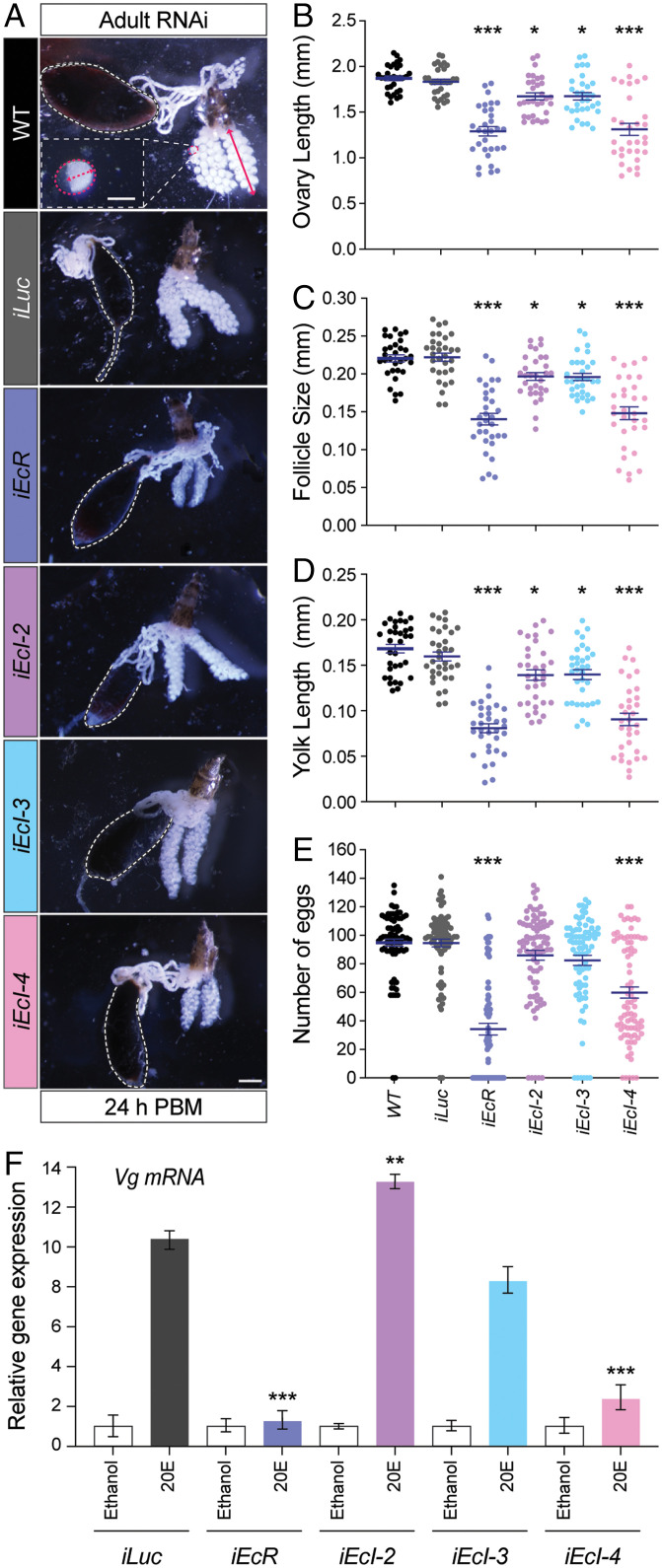
*EcI-4* is required for vitellogenesis in *Aedes* adult females. (*A*) Representative images of ovaries from wild-type (*WT*), *Luc* RNAi (*iLuc*; control), *EcR* RNAi (*iEcR*), and *EcI-2*, -*3*, and -*4* RNAi (*iEcI-2*, *iEcI-3*, and *iEcI-4*) females 24 h post blood meal (PBM). The gut filled with the blood meal is surrounded by dashed white lines. At the *Top*, ovary length, follicle size, and yolk size are indicated by a solid red arrow, dashed red line (longitudinal axis), and dashed red arrow, respectively. (Scale bars, 150 µm for follicle and 0.5 mm for ovary.) (*B*–*D*) Ovary length (*B*), follicle size (*C*), and yolk length (*D*) of wild-type (*WT*), *Luc* RNAi (*iLuc*; control), *EcR* RNAi (*iEcR*), and *EcI-2*, -*3*, and -*4* RNAi (*iEcI-2*, *iEcI-3*, and *iEcI-4*) females. All values are the means ± SEM from a minimum of three independent experiments with 10 individuals in each replicate. *P < 0.05, ***P < 0.001 from one-way ANOVA followed by Dunnett’s multiple comparison test as compared to control. (*E*) Number of deposited eggs per each individual of wild-type (*WT*), *Luc* RNAi (*iLuc*; control), *EcR* RNAi (*iEcR*), and *EcI-2*, -*3*, and -*4* RNAi (*iEcI-2*, *iEcI-3*, and *iEcI-4*) females. All values are the means ± SEM from a minimum of three independent experiments with 10 individuals in each replicate. ***P < 0.001 from one-way ANOVA followed by Dunnett’s multiple comparison test as compared to control. (*F*) Relative expression levels of *Vg* in the fat body dissected from *Luc* RNAi (*iLuc*; control), *EcR* RNAi (*iEcR*), and *EcI-2*, -*3*, and -*4* RNAi (*iEcI-2*, *iEcI-3*, and *iEcI-4*) females and cultured with or without 10 μM 20E in vitro, as assessed by qRT-PCR. All values are the means ± SEM (*n* = 4). **P < 0.01, ***P < 0.001 from one-way ANOVA followed by Dunnett’s multiple comparison test as compared to *iLuc* control.

We next injected dsRNA targeting each ecdysone importer or *EcR* into newly eclosed females, which were blood fed 3 d after injection. This resulted in ∼60 to 75% reduction of mRNA levels for each target gene (*SI Appendix*, Fig. S9). Vitellogenesis was clearly reduced in *EcR* RNAi animals as indicated by ovary length, follicle size, and yolk length (Fig. 5 *A*–*D*), consistent with the strong reduction of *Vg* expression in the fat body (*SI Appendix*, Fig. S9*E*). *Vg* expression was also strongly reduced in *EcI-4* RNAi animals, as compared to milder reduction caused by *EcI-2* or *EcI-3* knockdown (*SI Appendix*, Fig. S9*E*). Consistent with this, knockdown of *EcI-4*, but not *EcI-2* or *EcI-3*, caused similar reduction in vitellogenesis comparable to that caused by *EcR* knockdown (Fig. 5 *A*–*D*). As a result, both *EcR* RNAi and *EcI-4* RNAi caused significant reduction in fecundity (Fig. 5*E*).

Lastly, to further confirm that EcI-4 is important in the fat body to mediate ecdysone-induced yolk protein precursor synthesis, we performed in vitro culture experiments using the fat body dissected from RNAi animals. In the control fat body, 20E induced a robust, ∼10-time increase of *Vg* expression, which was almost completely blocked by *EcR* RNAi (Fig. 5*F*). Importantly, knockdown of *EcI-4*, but not *EcI-2* or *EcI-3*, caused a similar strong inhibition of 20E-induced *Vg* expression, suggesting a direct role of EcI-4 in ecdysone incorporation into the fat body. Taken together, our results indicate the predominant role of EcI-4 in ecdysone-mediated reproduction in adult female mosquitoes.

## Discussion

Recent studies in *Drosophila* have shown that ecdysone, the primary steroid hormone in insects, requires a membrane transporter EcI to enter its target cells and exert its genomic effects through EcR (6, 25). Although this critical function of EcI in ecdysone signaling seems to be highly conserved among other insect species (7), we noticed that *EcI* orthologs are unexpectedly missing in mosquitoes. In the present study, we characterized additional ecdysone importers, EcI-2, -3, and -4, in both *Drosophila* and *Aedes*. These additional ecdysone importers are dispensable for development and reproduction in *Drosophila*, likely reflecting the role of EcI as the predominant ecdysone importer. In contrast, in *Aedes* mosquitoes, EcI-2 is required for ecdysone-mediated developmental progression, whereas EcI-4 seems to be most critical for vitellogenesis in adult females. As these additional ecdysone importers show similar abilities to rescue *EcI* deficiency in flies, their differential roles in mosquito development and reproduction are most likely due to their distinct expression profiles as revealed in this study. Potential functions of EcI-3 in *Aedes* mosquitoes are currently unknown.

One of the remaining important questions yet to be fully addressed in ecdysone research is how different types of cells respond differently to a single steroid hormone, even when the cells express similar levels of the receptor. Previous studies have provided a few key answers to this question, including different EcR isoforms (28), cell-specific expression of different EcR cofactors (29, 30), and different chromatin accessibility among different cells (31). Our current study on additional ecdysone importers revealed another important molecular mechanism underlying this issue: These multiple transporters are under control of differential transcriptional regulation, allowing different types of cells to respond to ecdysone at different times. Investigation of such differential regulatory mechanisms of ecdysone importer expression is clearly warranted in future studies.

The evolutionary reason why *EcI* was lost in mosquito species is currently unknown. Our results nonetheless suggest that, in the absence of *EcI*, alternative ecdysone importers assume paramount importance in development and reproduction. This makes these additional ecdysone importers potential targets for development of mosquito-specific insect growth regulators and pesticides. The involvement of distinct ecdysone importers in development and reproduction may even make it possible to develop stage-specific regulators of ecdysone signaling in mosquitoes. The effort to identify efficient blockers of ecdysone importers is currently underway.

What are the functions of the additional ecdysone importers, particularly in insects that have *EcI* in the genome? Although *EcI-2*, -*3*, and -*4* are dispensable for *Drosophila* development and reproduction, it is conceivable that they have either unique or redundant functions that are not essential for overall viability and fertility under normal conditions. In this regard, it would be important to examine potential functions of these additional ecdysone importers in *Drosophila* and other insects more closely under different conditions, particularly in tissues where these transporters are highly expressed (6). Considering pleiotropic functions of ecdysone in insect physiology (1), it would also be interesting to investigate whether these additional ecdysone importers are involved in ecdysone functions that are not directly related to growth and reproduction, such as stress responses. Some tools developed in the current study, such as *EcI-2*, -*3*, and -*4* mutant flies, are expected to facilitate such future studies. Importantly, OATPs generally have a broad spectrum of substrates, and it has previously been reported, for example, that EcI-4 in the Malpighian tubules is involved in excretion of a plant-derived toxic substance in flies (32). It is also interesting to note here that a recent report identified a potential role of Oatp33Eb in promoting ecdysone uptake into cachectic tumors in *Drosophila* (33), although Oatp33Eb did not facilitate ecdysone uptake in our heterologous system. This may indicate functional interactions between multiple OATPs through trafficking of substrates other than ecdysteroids. When investigating as yet unknown functions of ecdysone importers, it is imperative to consider influences caused by transport of such additional substrates.

In conclusion, our results indicate unique functions of newly characterized ecdysone importers in development and reproduction in mosquitoes, which may pave the way for better control of these deadly human disease vectors. Functional characterization of ecdysone importers in different insect species is expected to deepen our understanding of how pleiotropic functions of ecdysone are regulated in different tissues under different conditions.

## Materials and Methods

### Flies.

All flies (*D. melanogaster*) were raised at 25 °C on standard fly food (6) under a 12-h light/dark cycle. *w^1118^* was used as a control strain. *EcI* mutant alleles (*EcI^1^* and *EcI^2^*) were generated previously (6); all the other ecdysone importer mutant alleles (*EcI-2^1^*, *EcI-3^1^*, *EcI-4^1^*, and *EcI-3–4^1^*) were generated using CRISPR/Cas9-mediated mutagenesis as described below. Deficiency alleles over ecdysone importer genes [*Df(2L)Exel6033*, #7516; *Df(2R)Exel7171*, #7902] and *armadillo* (*arm*)*-Gal4* (#1560) were obtained from the Bloomington *Drosophila* Stock Center (BDSC). *UAS-EcI-2*, *UAS-EcI-3*, and *UAS-EcI-4* were generated by subcloning full-length cDNA clones into *pUAST* vector (see below for details of cDNA clones). All new transgenic flies were generated by BestGene Inc.

### Mosquitoes.

The Rockefeller strain of *Ae. aegypti* was used in this study. Both male and female adults were maintained on 10% sucrose and reared at 27 °C and 80% relative humidity under a 16-h light/8-h dark cycle. Larvae in water were fed on a diet containing dry dog food (Blue Buffalo), fish flakes (Tetramin), and liver powder (Now Foods) (10:10:1 wt. ratio). All mosquito dissection was performed in 1× phosphate-buffered saline (PBS) solution (Fisher BioReagents) at room temperature. Using an artificial glass feeder, all adult female mosquitoes were allowed to feed on bovine blood purchased from Hemostat. Only fully engorged female mosquitoes were used.

### Comprehensive Identification of OATPs in Dipteran Species.

Using amino acid sequences of all eight *Drosophila* OATPs as queries, whole-genome sequences of the housefly *Musca domestica*, the sand fly *P. papatasi*, the yellow fever mosquito *Ae. aegypti*, the African malaria mosquito *An. gambiae*, and the southern house mosquito *C. quinquefasciatus* were screened (TBLASTN analysis) using NCBI BLAST (https://blast.ncbi.nlm.nih.gov/Blast.cgi) and VectorBase BLAST (https://vectorbase.org/vectorbase/app/search/transcript/UnifiedBlast). All the obtained sequences were reciprocally screened against *Drosophila* proteome (BLASTP analysis) by using FlyBase BLAST (https://flybase.org/blast/) to confirm their orthologous relationships with *Drosophila* OATPs. This two-step screening identified eight OATPs in *M. domestica*, eight OATPs in *P. papatasi* (two of which are likely pseudogenes), six OATPs in *Ae. aegypti*, six OATPs in *An. gambiae*, and seven OATPs in *C. quinquefasciatus*. Protein names and GenBank accession numbers are listed in *SI Appendix*, Table S1. The sequence similarities between *Aedes* and *Drosophila* ecdysone importers were calculated using the EMBOSS Water Pairwise Sequence Alignment tool (https://www.ebi.ac.uk/Tools/psa/emboss_water/) (34).

### Phylogenetic Tree Analysis.

The unrooted neighbor-joining tree (Fig. 1*A*) was generated using ClustalW (https://www.ddbj.nig.ac.jp/services/clustalw-e.html) (35, 36). Entire amino acid sequences of all OATPs in the dipteran species mentioned above (*SI Appendix*, Table S1) were aligned. Bootstrap analyses of 1,000 replications were conducted to assess the relationships.

### Cloning of OATP-Encoding Genes.

For *Drosophila* OATP-encoding genes, full-length cDNA clones from the *Drosophila* Genomics Resource Center (*EcI-2/Oatp33Ea*, LD36578; *EcI-3/Oatp58Dc*, LP09443; *Oatp58Da*, IP17768; *Oatp33Eb*, RE09129; *Oatp26F*, RE32029; and *Oatp30B*, RE26532) were subcloned into the *pcDNA3.1* vector. The coding sequence of *EcI-4/Oatp58Db* was PCR amplified from whole-body *Drosophila* cDNA using Phusion Plus DNA Polymerase (Thermo Fisher Scientific) with the primers listed in *SI Appendix*, Table S4. The PCR product was cloned into the *pcDNA3.1* vector and sequenced.

For *Aedes* OATP-encoding genes, total RNA was collected from fourth instar larvae using TRIzol reagent (Invitrogen) according to the manufacturer’s instructions. cDNA was generated from purified total RNA using PrimeScript RT Master Mix (Takara Bio). PCR amplification of the sequence corresponding to *EcI-2/AAEL007691*, *EcI-3/AAEL010921*, *EcI-4/AAEL010917*, *AAEL001812*, *AAEL001808*, and *AAEL007693* was performed using Phusion Plus DNA Polymerase with the primers listed in *SI Appendix*, Table S5. The PCR products were cloned into the *pcDNA3.1* vector and sequenced.

### Transfection and Luciferase Reporter Assay in HEK293 Cells.

HEK cells (obtained from Michael E. Adams, University of California, Riverside, CA) at a density of 4 × 10^5^ cells/mL were seeded in 100 µL/well of Opti-MEM reduced serum media (Thermo Fisher Scientific) containing 5% fetal bovine serum (FBS) and 1% MEM nonessential amino acids (NEAA) solution (Thermo Fisher Scientific) in a 96-well clear flat bottom microplate (Corning). Transfection of HEK293 cells was performed using Attractene transfection reagent (Qiagen) by the fast-forward transfection approach following the manufacturer’s instructions. A total of 50 µL/well of transfection mixture containing Opti-MEM reduced serum media, Attractene, and DNA plasmids was added to each well, bringing the final volume to 150 µL/well. A total of 0.1 µg/well of *pcDNA3.1* empty vector (control) or *pcDNA3.1* vector containing full-length cDNA of *Drosophila* or *Aedes* OATP-encoding genes was transfected, along with 60 ng/well of *pERV3* receptor plasmid (Agilent Technologies) containing a modified ecdysone receptor (VgEcR) and RXR, 36 ng/well of *pEGSH-LUC* luciferase reporter plasmid (Agilent Technologies), and 0.9 ng/well of *pRL-CMV* Renilla luciferase reporter plasmid (Promega) as a reference. After 24 h of incubation at 37 °C and 5% CO_2_, transfection medium was removed and replaced with 150 µL/well of Dulbecco's Modified Eagle Medium (DMEM) with 4.5 mg/mL glucose and sodium pyruvate without L-glutamine and phenol red (w-G-SP, wo-G-PR) (Thermo Fisher Scientific) containing 10% FBS, 1% Penicillin-Streptomycin Solution (PSS), and 1% MEM NEAA solution. After 48 h of incubation at 37 °C and 5% CO_2_, medium was removed and replaced with 150 µL/well of DMEM (w-G-SP, wo-G-PR) containing 1% PSS, 1% MEM NEAA solution, and 20E at indicated concentrations. After 24 h of incubation at 37 °C and 5% CO_2_, 75 µL of media was removed (total 75 µL remaining per well) and 75 µL/well of Dual-Glo Solution was added (total 150 µL/well). After 20 min of incubation at room temperature in the dark, 120 µL/well of cell lysates were transferred to 96-well solid white flat bottom polystyrene TC-treated plates. The firefly luciferase activity and cotransfected Renilla luciferase activity were measured subsequently using the Dual-Luciferase Reporter Assay System in accordance with the manufacturer’s instructions and analyzed with GloMax-Multi + Microplate Multimode Reader with Instinct. For each condition, cells were treated independently in three wells of a 96-well plate in each experiment, and the same experiment was conducted multiple times on different days.

### Total RNA Extraction and qRT-PCR.

Animals or dissected tissues were collected in 1.5-mL tubes and immediately flash-frozen in liquid nitrogen. Total RNA from animals or tissues was extracted using TRIzol reagent (Invitrogen) according to the manufacturer’s instructions. Extracted RNA was further purified by RNeasy mini kit (Qiagen) following the manufacturer’s instructions, combined with treatment with RNase-Free DNase Set (Qiagen). cDNA was generated from purified total RNA using PrimeScript RT Master Mix (Takara Bio). qRT-PCR was performed on the CFX connect real-time PCR detection system (Bio-Rad) using SYBR Premix Ex Taq II (Tli RNaseH Plus) (Takara Bio). For absolute quantification of mRNAs, serial dilutions of pGEM-T (Promega) plasmids containing coding sequences of the target genes or internal control genes (*rp49* for *D. melanogaster* and *AeRpL32* (*AAEL003396*) for *Ae. aegypti*) were used as standards. After the molar amounts were calculated, transcript levels of the target mRNA were normalized to internal control gene levels in the same samples. Three to four separate samples were collected for each experiment and duplicate measurements were conducted. The primers used are listed in *SI Appendix*, Tables S4 and S5.

### *EcI* Mutant Rescue Experiments in *Drosophila.*

*EcI* mutant rescue experiments were conducted by weak ubiquitous expression of *UAS-EcI-2*, -*3*, and -*4* transgenes, respectively, in the *EcI* mutant background. *arm-Gal4; EcI^1^/TM6b-Dfd-EGFP* flies were crossed to either *w^1118^* (heterozygous mutant control), *EcI^2^/TM6b-Dfd-EGFP* (transheterozygous mutant control), *UAS-EcI; EcI^2^/TM6b-Dfd-EGFP* (positive control), *UAS-EcI-2; EcI^2^/TM6b-Dfd-EGFP*, *UAS-EcI-3; EcI^2^/TM6b-Dfd-EGFP*, or *UAS-EcI-4; EcI^2^/TM6b-Dfd-EGFP*, respectively. Eggs were laid on grape juice plates with yeast paste at 25 °C for 6 h. After 24 h, early first instar larvae just after hatching were collected (homozygous mutant larvae were collected by selecting those without the balancer chromosome with GFP) and transferred into vials with standard food (fewer than 50 animals/vial). Developmental stages were scored at 36 h and 120 h after hatching by checking stage-specific morphology of larval mouth hooks and posterior spiracles (6).

### Generation of *EcI-2*, *EcI-3*, and *EcI-4* Mutants in *Drosophila.*

*EcI-2*, *EcI-3*, and *EcI-4* mutant alleles were generated using the CRISPR/Cas9 system. Pairs of gRNA target sequences (20 bp: T1 and T2) were designed near the transcription start and stop sites of each target gene using NIG-FLY Cas9 Target finder (NIG) (*SI Appendix*, Fig. S2). T1 from *EcI-3* and T2 from *EcI-4* were used for making the *EcI-3–4* double mutant. Forward and reverse 24-bp oligonucleotides with 20-bp target sequences (Table S4) were annealed to generate a double-strand DNA with 4-bp overhangs on both ends and inserted into *Bbs*I-digested *pBFv-U6.2* or *pBFv-U6.2B* vector provided by the NIG (37). To construct double-gRNA vectors, the first gRNA (T1) was cloned into *pBFv-U6.2* (named *pBFv-U6.2-T1*), whereas the second gRNA (T2) was cloned into *pBFv-U6.2B* (named *pBFv-U6.2B-T2*). A fragment containing the U6 promoter, and the first gRNA was cut out from *pBFv-U6.2-T1* and ligated into *pBFv-U6.2B-T2* (named *pBFv-U6.2B-T1-T2*). These double-gRNA vectors (*pBFv-U6.2B-T1-T2*) were independently injected into embryos of *yw;; nos-cas9 (III-attP2)/TM6B* flies (BestGene Inc).

For each target gene, surviving G_0_ males were divided into 10 groups and crossed en masse to *Sp/CyO-GFP* (obtained from Takashi Nishimura) virgin female flies. From the progeny of each of these 10 crosses, five single males were isolated and crossed independently to *Sp/CyO-GFP* virgin female flies to establish independent isogenized lines. To confirm deletions of the target genes, we extracted genome DNA from these 50 lines using the DNA extraction buffer (10 mM Tris⋅HCl, 1 mM Ethylenediaminetetraacetic acid, 25 mM NaCl, and 200 µg/mL Proteinase K), and PCR amplification was performed by using primers listed in *SI Appendix*, Table S4. Out of 50 lines, several lines from each target pair possessed deletion mutations. The PCR products were sequenced, and complete deletions were confirmed in all lines. We selected one mutant allele for each target gene and named them as *EcI-2^1^*, *EcI-3^1^*, *EcI-4^1^*, and *EcI-3–4^1^*. *EcI-2^1^* has a 3,704-bp deletion including the 5′ untranslated region and almost the entire *EcI-2/Oatp33Ea* coding sequence (CDS) (*SI Appendix*, Fig. S2*A*). *EcI-3^1^* has a 3,535-bp deletion including the 5′ untranslated region and almost the entire *EcI-3/Oatp58Dc* CDS (*SI Appendix*, Fig. S2*B*). *EcI-4^1^* has two deletions with a 9-bp deletion including the transcriptional start site and a 2,092-bp deletion including almost the entire *EcI-4/Oatp58Db* CDS (*SI Appendix*, Fig. S2*C*). *EcI-3–4^1^* has a 6,495-bp deletion including the 5′ untranslated region of *EcI-3/Oatp58Dc*, the entire *EcI-3/Oatp58Dc* CDS, and almost the entire *EcI-4/Oatp58Db* CDS (*SI Appendix*, Fig. S2*D*).

### Pupariation Timing Analysis in *Drosophila.*

Flies were allowed to lay eggs on 3% agar plates containing 30% grape juice (Welch’s) at 25 °C. Newly hatched larvae were transferred into vials with mashed standard food (25 larvae/vial), and pupae were counted at indicated time points. For each genotype, four vials were scored two times a day.

### Pupal Volume Measurements in *Drosophila.*

Pupal length and width were determined from images captured with a Zeiss Axiocam 506 color digital camera attached to a SteREO Discovery.V12 microscope (Zeiss). Images were processed using ImageJ 1.53v (NIH). The pupal volume was determined by the following approximate equation: 4/3π (length × width^2^).

### Egg Laying Assay in *Drosophila.*

Four-day-old single virgin females and five to six males were transferred to a vial with standard food to allow mating and egg laying. After 24 h, individual females were then transferred to a fresh vial for further egg laying for another 24 h. The total number of eggs laid in 2 d (48 h) was counted.

### Mutagenesis in *Aedes.*

sgRNAs were designed immediately downstream of the predicted start codon of each ecdysone importer gene (*SI Appendix*, Fig. S4). One guanine was added at the 5′ terminal end of each sgRNA to facilitate transcription by T7 RNA polymerase (*SI Appendix*, Table S5). Potential off-target binding was checked by using two online tools: zifit.partners.org/ZiFiT/andcrispr.mit.edu. dsDNA templates for sgRNA synthesis were generated by template-free PCR, using a specific forward primer for each gene and one universal reverse primer (*SI Appendix*, Table S5). sgRNA was synthesized using the MEGAscript T7 Transcription Kit (Ambion) and purified using the MEGAclear Transcription Clean-Up Kit (Ambion) following the manufacturer’s protocols. A mixture of sgRNA (40 ng/μL) and Cas9 protein with a nuclear localization signal (PAN Bio; 300 ng/μL) was microinjected into the posterior pole of preblastoderm embryos at an angle of 10 to 25°. The sgRNA/Cas9 ratio was optimized according to a previously described protocol (38). The embryos were hatched 5 d postinjection and reared thereafter as described above. The microinjection was repeated three times for a total of 450 embryos per each gene.

The genomic DNA was extracted from newly hatched larvae using the DNA extraction buffer. Fragments containing sgRNA target sites were amplified using gene-specific primers (*SI Appendix*, Table S5). The PCR products were gel purified using the Gel DNA Recovery Kit (Zymoclean), cloned into the pGEMT-easy vector (Thermo Fisher Scientific), and sequenced. CRISPR-Cas9 mutagenesis efficiency was assessed through a T7 Endonuclease 1 (T7E1) assay. In brief, 200 ng of PCR products in 1× NEB buffer 2 (New England Biolabs) were hybridized under the following conditions: 95 °C for 5 min, 95 to 85 °C at −2 °C/s, 85 to 25 °C at −1 °C/s, and held at 4 °C. Ten units of T7E1 enzyme (New England Biolabs) were added to each sample, and the samples were incubated at 37 °C for 15 min. Products were visualized using 2% agarose gel electrophoresis.

### dsRNA Synthesis for RNAi in *Aedes.*

For dsRNA synthesis, a T7 promoter sequence, TAATACGACTCACTATAGGGAGA, was added to the 5′ end of each primer as listed in *SI Appendix*, Table S5. PCR was performed using the OneTaq-Quick-Load 2× Master Mix (New England BioLabs) with *Aedes* whole-body cDNA as a template, and the amplified PCR products were cleaned using the Gel DNA Recovery Kit (Zymoclean). dsRNA was synthesized using the MEGAscript T7 Transcription Kit (Ambion).

### RNAi in *Aedes* Larvae.

Prior to hatching, mosquito eggs were allowed to develop for a minimum of 1 wk. To collect newly hatched larvae, eggs were submerged in a container with water and food and left at room temperature overnight. On the following day, the container was moved into the incubator set at 27 °C and 80% relative humidity. Twenty newly hatched larvae were transferred into a 1.5-mL Eppendorf tube containing 100 µL of premixed food and dsRNA (0.5 µg/µL final concentration). Larvae were soaked in the dsRNA solution for 4 h, after which they were transferred into a 1-oz cup containing 4 mL of premixed food. All surviving larvae were periodically monitored and counted thereafter. The head capsule length (head size) and siphon length were measured using images captured with a Zeiss Axiocam 506 color digital camera attached to a SteREO Discovery.V12 microscope (Zeiss).

For 20E and CF rescue experiments, larvae were cultured in 20E (Sigma-Aldrich; final concentration of 1 µM in 0.004% ethanol), CF (Sigma-Aldrich; final concentration of 10 nM in 0.00002% ethanol), or 0.004% ethanol (control) from 22 h after hatching, and surviving larvae were periodically monitored and counted thereafter.

### RNAi in *Aedes* Adult Females.

Purified dsRNA was resuspended with high-performance liquid chromatography-grade water (Thermo Fisher Scientific) at 5 μg/μL. Newly emerged virgin females were separated using a manual aspirator into a new cage and provided with 10% sucrose. Three days after eclosion, virgin females were anesthetized on ice and injected with 2.0 μg dsRNA (400 nL) using a Nanoject II microinjector (Drummond Scientific Company). After injection, females were placed back in a cage with 10% sucrose to recover for 24 h at 27 °C with 80% humidity.

### Ovary Length, Follicle Size, and Yolk Length Measurements in *Aedes* Adult Females.

After postinjection recovery, adult females were allowed to mate for 48 h and then allowed to feed on bovine blood for 1 h. Fully engorged females were selected and isolated in a cage and provided with 10% sucrose. At 24 h post blood meal, dsRNA-injected females were anesthetized for 5 min at −20 °C. Ovaries were then dissected, and the length of the central longitudinal axis of the ovary was measured. The ovary length of each female was calculated as an average length of the two ovaries. For primary follicle size measurement, the length of the longitudinal axis of 15 primarily follicles per ovary was measured, and the average follicle length of each female was calculated. The yolk length of each female was determined by measuring the average yolk length of all oocytes from one ovary. All measurements were conducted using images captured with a Zeiss Axiocam 506 color digital camera attached to a SteREO Discovery.V12 microscope (Zeiss). Images were processed using ImageJ 1.53v (NIH).

### Egg Laying Assay in *Aedes.*

After postinjection recovery, adult females were allowed to mate for 48 h and then allowed to feed on bovine blood for 1 h. Fully engorged females were selected, and individual females were placed in a cup with oviposition paper. The number of eggs was counted at 96 h post blood meal.

### In Vitro *Aedes* Fat Body Culture.

The fat body culture medium was prepared as previously described (39), with the TES buffer prepared as described in ref. 40. In vitro fat body culture experiments were performed as reported previously (39–41). Fat bodies dissected from five adult females 2 d after dsRNA injection were incubated in a single well of 96-well plates for 6 h in the culture medium with either 10 μM 20E or solvent (0.04% ethanol). After incubation, fat bodies were harvested, and total RNA extraction and qRT-PCR were performed as described above.

## Supplementary Material

Supplementary File

## Data Availability

All study data are included in the article and/or *SI Appendix*.
