# Supplementary material for: Essential functions of mosquito ecdysone importers in development and reproduction
Source: Proc Natl Acad Sci U S A. 2022 Jun 13;119(25):e2202932119. doi: 10.1073/pnas.2202932119 (PMC9231622; doi:10.1073/pnas.2202932119)
Supplement: Supplementary File [file pnas.2202932119.sapp.pdf]

Supplementary Information

Essential functions of mosquito ecdysone importers in development and reproduction

Lewis V. Hun, Naoki Okamoto, Eisuke Imura, Roilea Maxson, Riyan Bittar, Naoki Yamanaka*

*Correspondence: naoki.yamanaka@ucr.edu

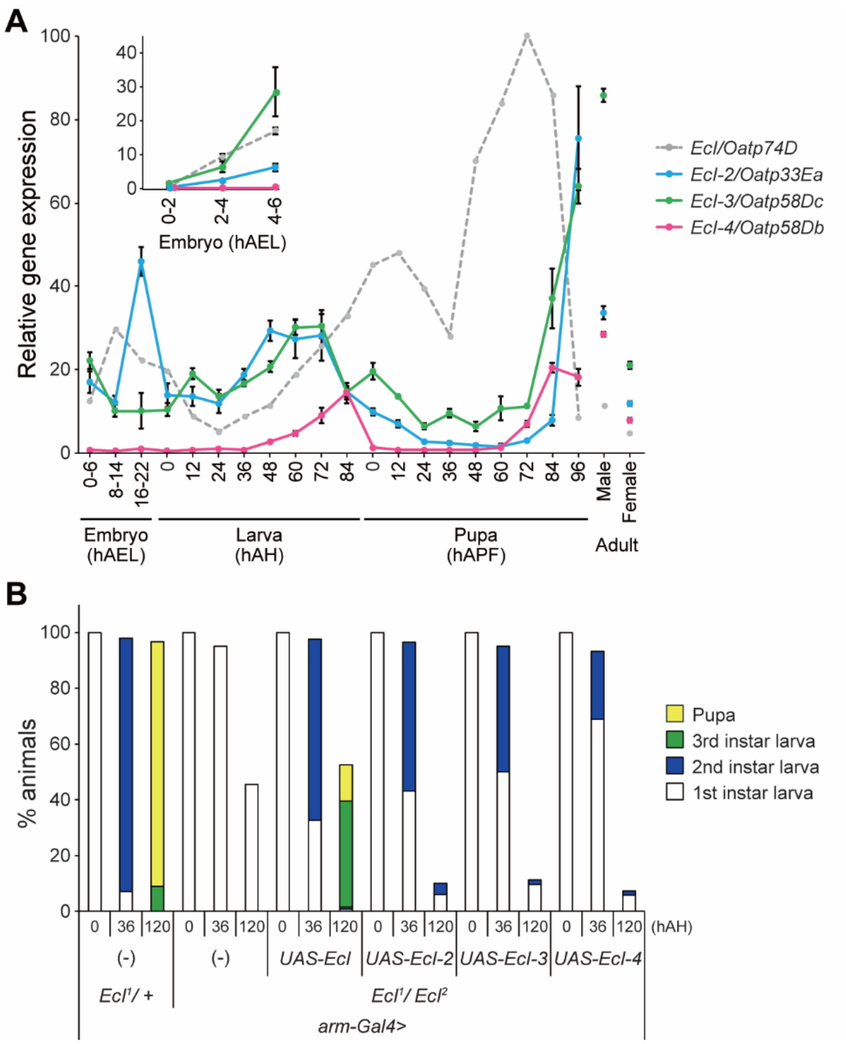

Figure S1. Expression levels and *in vivo* functions of ecdysone importer genes in *Drosophila*.

(A) Relative expression levels of ecdysone importer genes in the whole body during *Drosophila* development, as assessed by qRT-PCR. Samples were collected from w^{1118} animals. *EcI* expression levels are adopted from Okamoto et al. (6), and values are shown as percentages relative to the highest expression level of *EcI*. hAEL, hours after egg laying; hAH, hours after hatching; hAPF, hours after puparium formation. Adult cDNA samples were prepared from flies at 24 hr after eclosion. All values are the means ± SD (n = 3).

(B) Developmental progression and survival rate (%) of *EcI* heterozygous mutant control (*EcI*^{1}/+) and *EcI* transheterozygous mutant (*EcI*^{1}/*EcI*^{2}) rescued by weak ubiquitous expression of four different ecdysone importers (*arm-Gal4 > UAS-ecdysone importer; EcI*^{1}/*EcI*^{2}). Color bars indicate percentages of first instar larvae (white), second instar larvae (blue), third instar larvae (green), and prepupae/pupae (yellow).

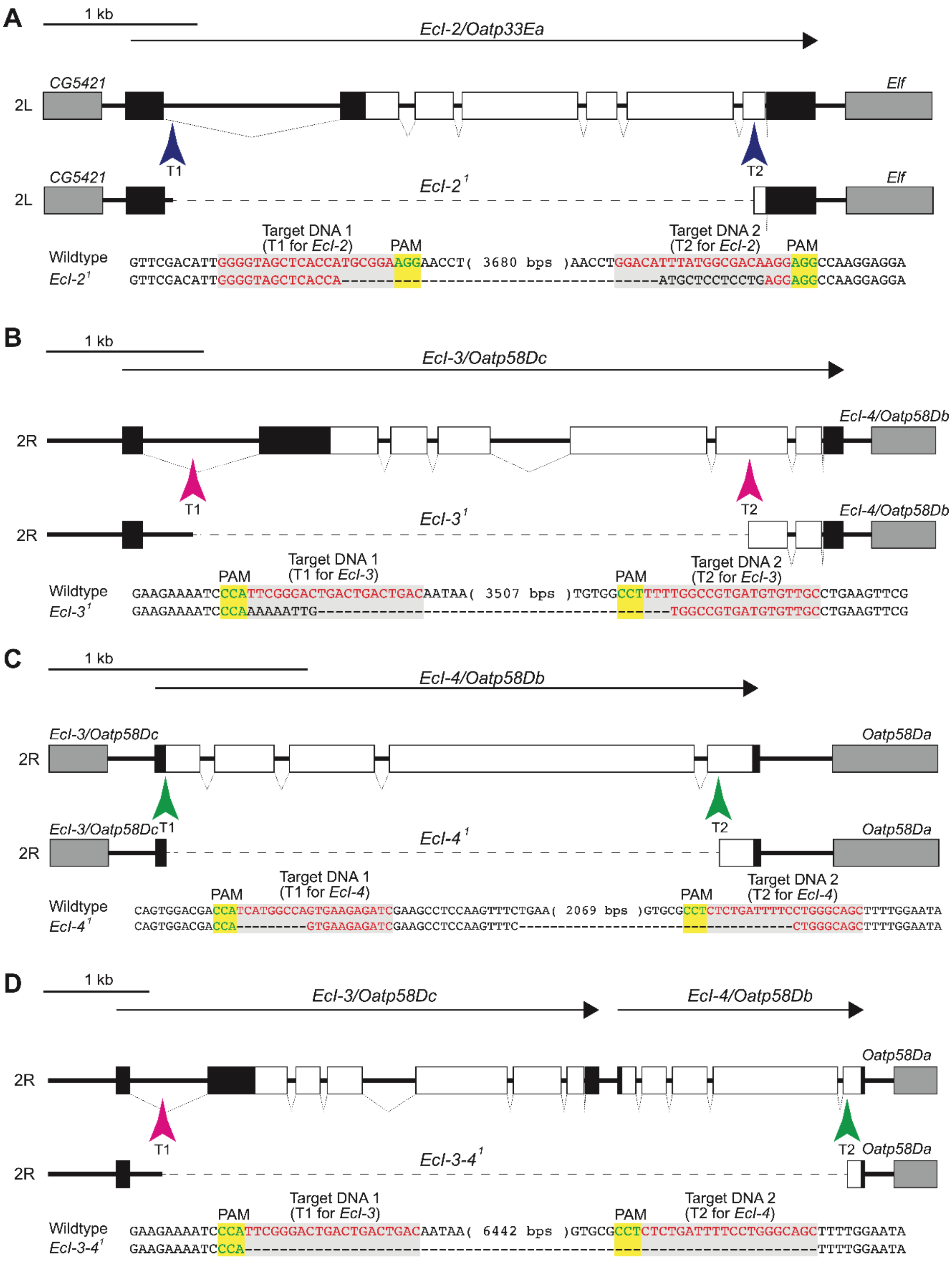

Figure S2. Generation of ecdysone importer mutants in *Drosophila* using CRISPR/Cas9.

(A-D) Schematic representation of the guide RNA (gRNA) targets for generating *EcI-2* (A), *EcI-3* (B), *EcI-4* (C), and *EcI-3-4* double (D) mutants in *Drosophila*. The protein-coding DNA sequences (CDSs) and untranslated regions are represented by open and filled boxes, respectively. Neighboring genes are represented by gray boxes. Arrows indicate the orientation of ecdysone importer genes, while arrowheads (T1 and T2) indicate gRNA target sequences. Sequences of ecdysone importer mutants as compared to the wildtype sequences are shown at the bottom of each panel. gRNA target sequences are shown in red, and the neighboring NGG protospacer adjacent motif (PAM) sequences are shown in green. Deleted residues are shown as dashes.

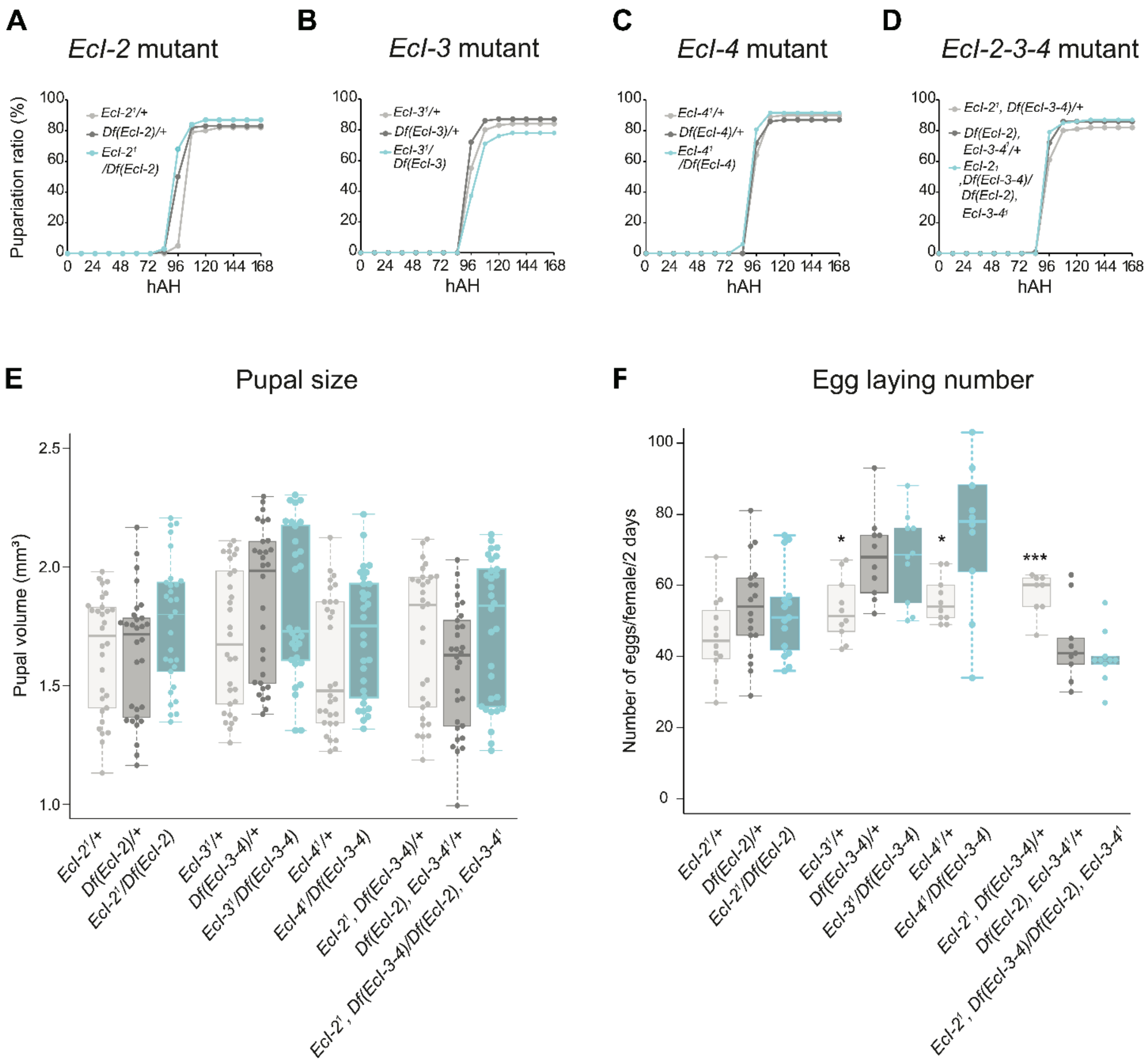

Figure S3. Growth and reproductive phenotypes of ecdysone importer mutants in *Drosophila*.

(A-D) Pupariation timing of *Ecl-2* (A), *Ecl-3* (B), *Ecl-4* (C), and *Ecl-2-3-4* triple (D) mutants in *Drosophila*. Df indicates deficiency alleles over ecdysone importer genes indicated in parenthesis. Blue lines indicate transheterozygous ecdysone importer mutants, whereas gray lines indicate heterozygous controls. hAH, hours after hatching. n = 100 from 4 independent experiments.

(E, F) Box plot of pupal size (E) and egg laying number (F) of transheterozygous ecdysone importer mutants (blue) and heterozygous controls (gray). Pupal size is shown as pupal volume (mm^3) calculated using the length and width of the pupae (n = 30 from 4 independent experiments), whereas egg laying number is shown as the number of eggs laid per female every two days (n = 10-18 from 2 independent experiments). Although there was some variability in laid egg numbers among heterozygous controls, none of the individual nor combination mutants showed any discernible defects. *$p < 0.05$, ***$p < 0.001$ from Mann-Whitney U test with Bonferroni Correction.

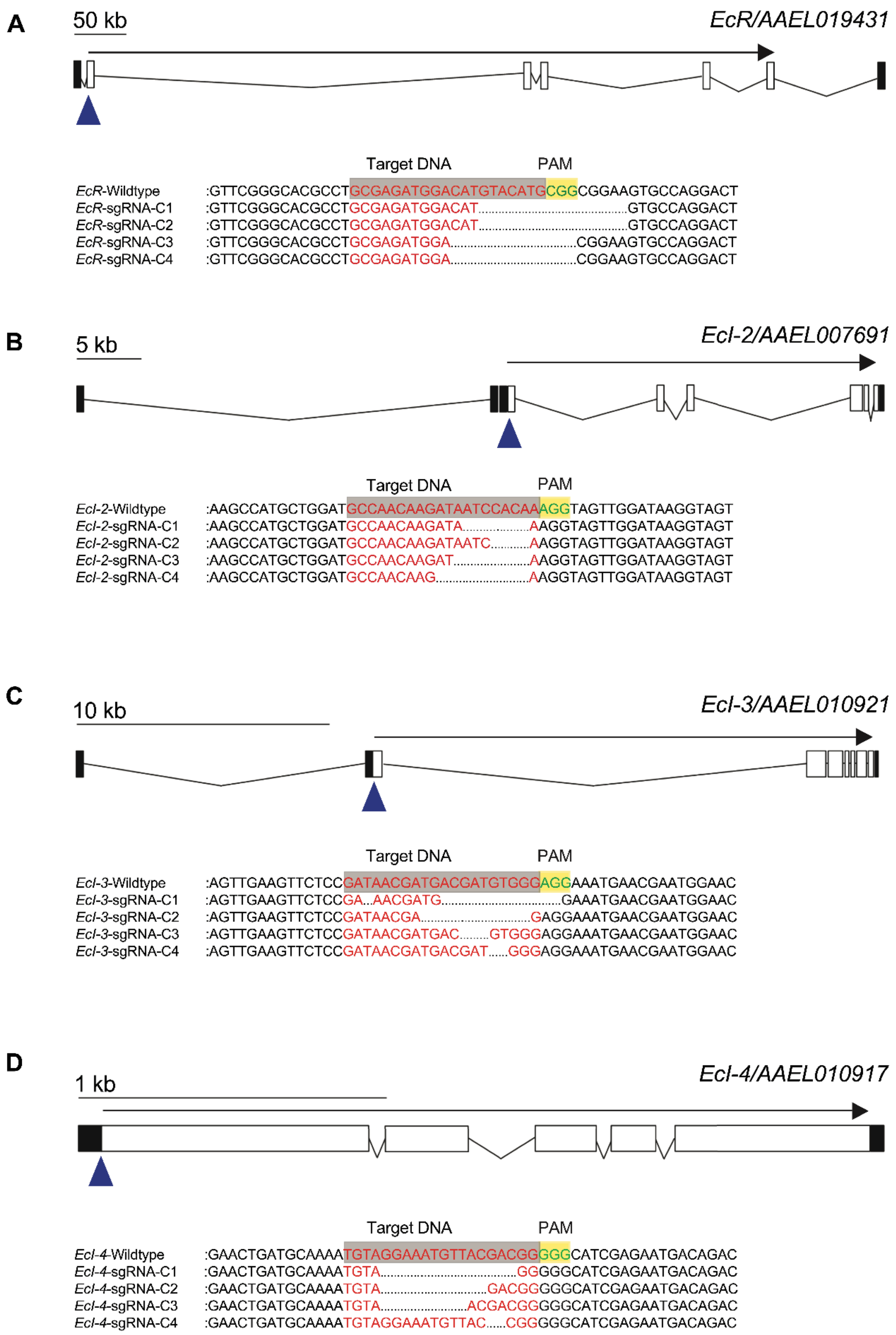

Figure S4. Generation of *EcR* and ecdysone importer mutants in *Aedes* using CRISPR/Cas9.

(A-D) Schematic representation of the guide RNA (gRNA) targets for generating *EcR* (A), *EcI-2* (B), *EcI-3* (C), and *EcI-4* (D) mutants in *Aedes*. The protein-coding DNA sequences (CDSs) and untranslated regions are represented by open and filled boxes, respectively. Arrows indicate the orientation of the genes, while arrowheads indicate gRNA target sequences. Sequences of the mutants from 4 independent PCR-amplified clones are shown as compared to the wildtype sequences at the bottom of each panel. gRNA target sequences are shown in red, and the neighboring NGG protospacer adjacent motif (PAM) sequences are shown in green. Deleted residues are shown as dotted lines.

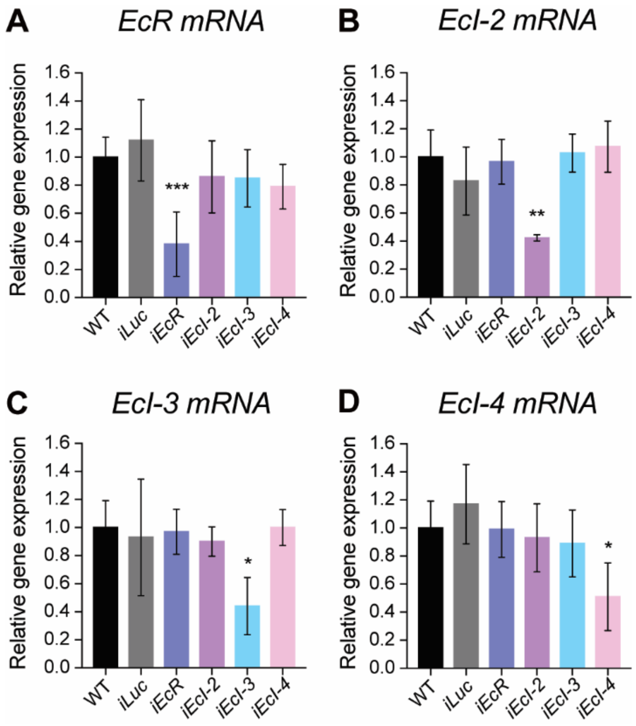

Figure S5. Knockdown efficiency and specificity of dsRNA soaking experiments in *Aedes* larvae.

(A-D) Relative expression levels of *EcR* (A), *EcI-2* (B), *EcI-3* (C), and *EcI-4* (D) in WT (wildtype; control), *Luc* RNAi (*iLuc*; control), *EcR* RNAi (*iEcR*), and *EcI-2*, *3*, and *4* RNAi (*iEcI-2*, *iEcI-3*, and *iEcI-4*) animals at 48 hours after hatching, as assessed by qRT-PCR. All values are the means ± SEM (n = 4). *p < 0.05, **p < 0.01, ***p < 0.001 from one-way ANOVA followed by Dunnett's multiple comparison test as compared to *iLuc* control.

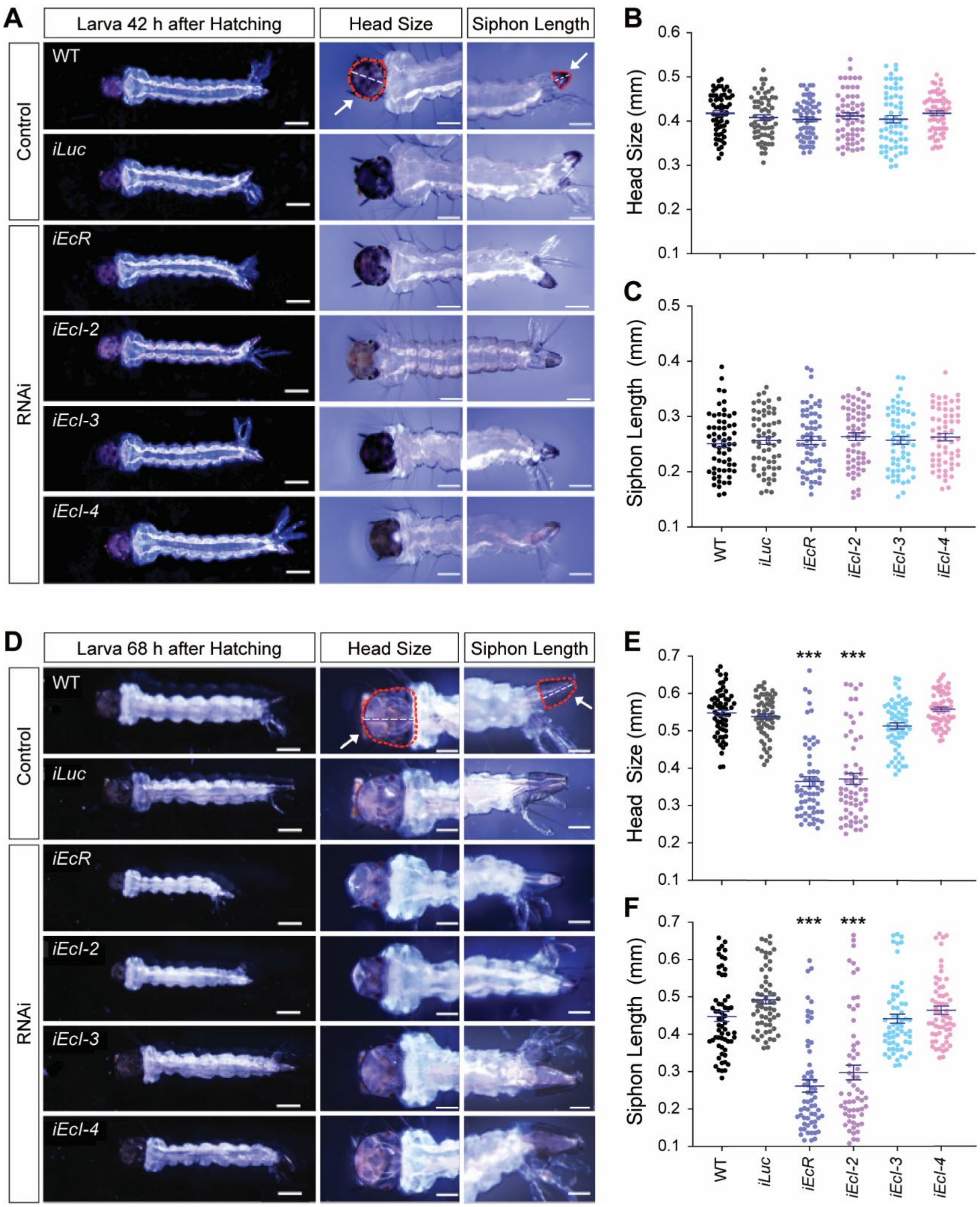

Figure S6. Detailed developmental phenotype of dsRNA-treated *Aedes* larvae.

(A-F) Representative images of the whole body, head, and siphon of dsRNA-treated larvae and their measurements at 42 hrs (A-C) or 68 hrs (D-F) after hatching. WT, wildtype (control); *iLuc*, *Luc* RNAi (control); *iEcR*, *EcR* RNAi; *iEcl-2*, *Ecl-2* RNAi; *iEcl-3*, *Ecl-3* RNAi; *iEcl-4*, *Ecl-4* RNAi. Scale bars, 0.5 mm for whole larva images; 250 μm for head and siphon images. The head and siphon are circled with red dashed lines and marked with white arrows in the WT images in (A) and (D). The head capsule length (head size) and siphon length (indicated by white dashed lines in the same images) were measured in (B), (C), (E), and (F). All values are the means ± SEM (n = 60 from 3 independent experiments). ***p < 0.001 from one-way ANOVA followed by Dunnett's multiple comparison test as compared to *iLuc* control.

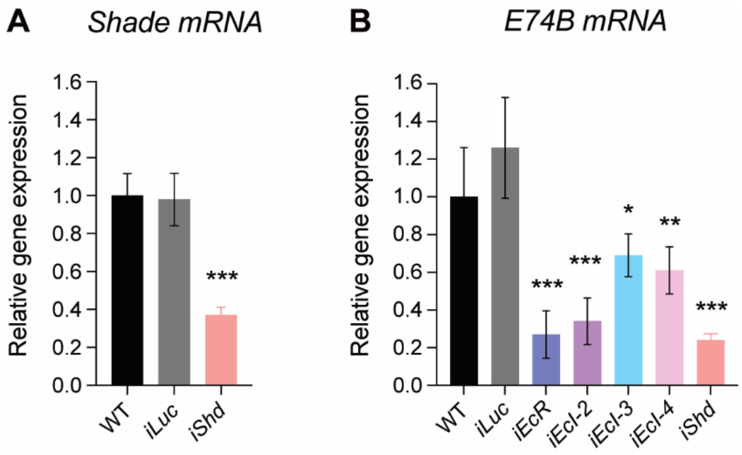

Figure S7. *Shade* and *E74B* expression levels in dsRNA-treated *Aedes* larvae.

(A) Relative expression levels of *Shade* in WT (wildtype; control), *Luc* RNAi (*iLuc*; control), and *Shade* RNAi (*iShd*) animals at 48 hours after hatching, as assessed by qRT-PCR. All values are the means ± SEM (n = 3). ***p < 0.001 from one-way ANOVA followed by Dunnett's multiple comparison test as compared to *iLuc* control.

(B) Relative expression levels of *E74B* in WT (wildtype; control), *Luc* RNAi (*iLuc*; control), *EcR* RNAi (*iEcR*), *EcI-2*, *3*, and *4* RNAi (*iEcI-2*, *iEcI-3*, and *iEcI-4*), and *Shade* RNAi (*iShd*) animals at 48 hrs after hatching, as assessed by qRT-PCR. All values are the means ± SEM (n = 4). *p < 0.05, **p < 0.01, ***p < 0.001 from one-way ANOVA followed by Dunnett's multiple comparison test as compared to *iLuc* control.

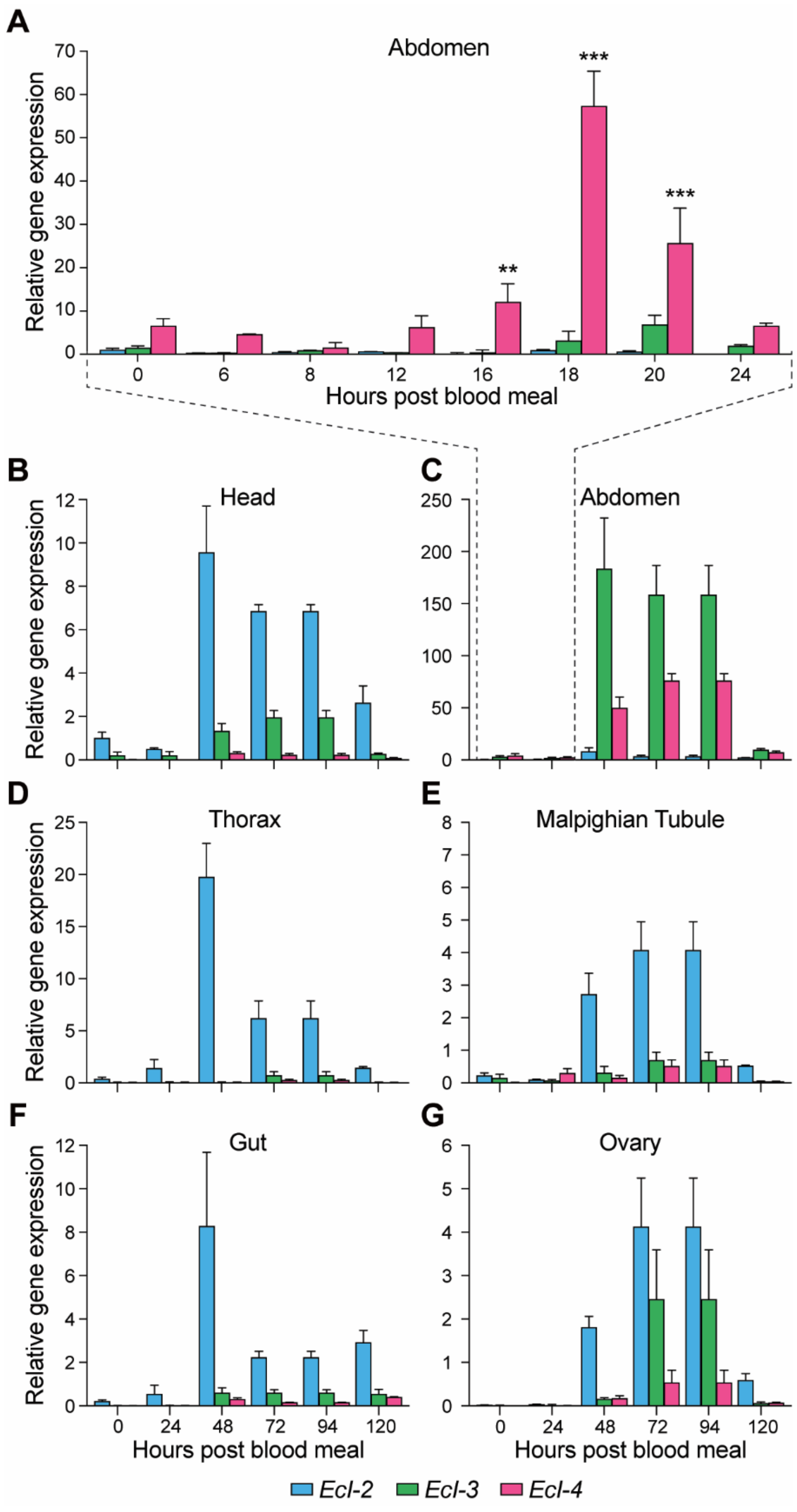

Figure S8. Ecdysone importer gene expression in *Aedes* adult females after blood meal.

(A) Relative expression levels of *EcI-2*, *3*, and *4* in the abdomen during the first 24 hours post blood meal, as assessed by qRT-PCR. Values are shown relative to the *EcI-2* level in the head at 0 hour post blood meal in (B). All values are the means ± SEM (n = 3). $**p < 0.01$, $***p < 0.001$ from one-way ANOVA followed by Bonferroni's multiple comparison test among samples at the same time point.

(B-G) Relative expression levels of *EcI-2*, *3*, and *4* in the head (B), abdomen (C), thorax (D), Malpighian tubule (E), gut (F), and ovary (G), as assessed by qRT-PCR. Values are shown relative to the *EcI-2* level in the head at 0 hour post blood meal. All values are the means ± SEM (n = 5).

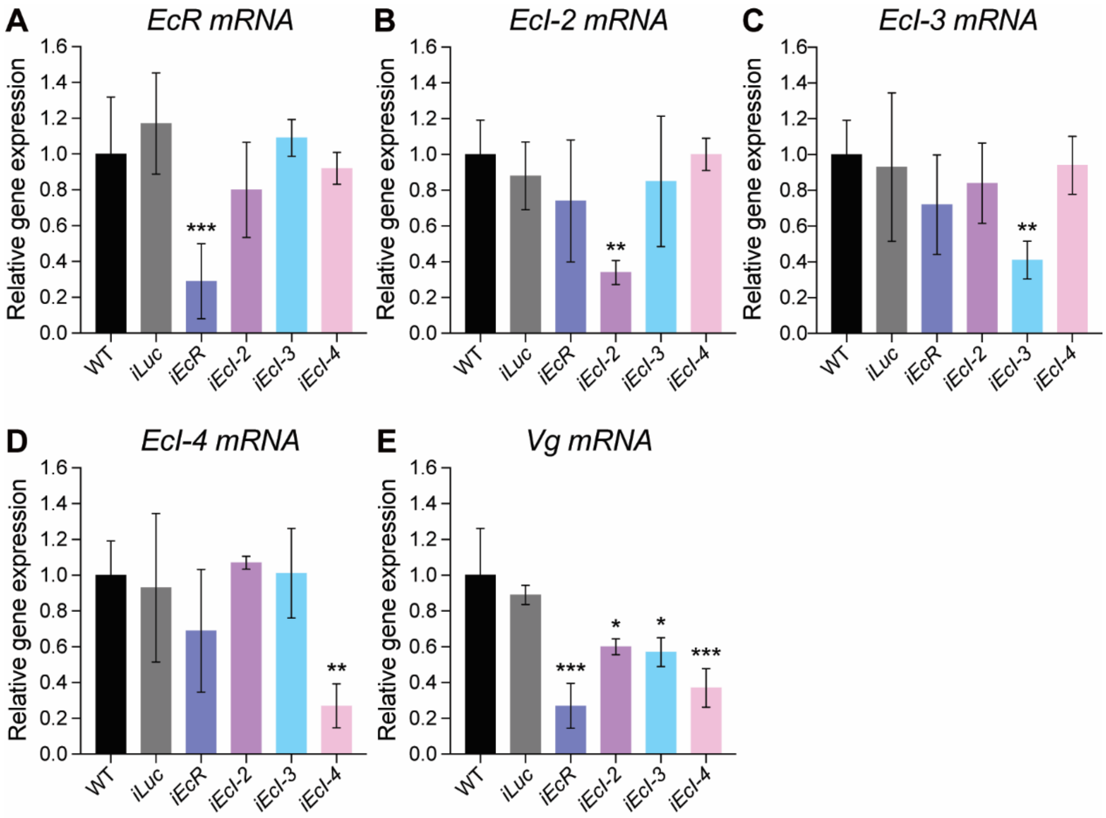

Figure S9. Gene expression levels in dsRNA-injected *Aedes* adult females.

(A-E) Relative expression levels of *EcR* (A), *EcI-2* (B), *EcI-3* (C), *EcI-4* (D), and *Vg* (E) in the fat body (abdominal wall with adhered fat body) of WT (wildtype; control), *Luc* RNAi (*iLuc*; control), *EcR* RNAi (*iEcR*), and *EcI-2*, *3*, and *4* RNAi (*iEcI-2*, *iEcI-3*, and *iEcI-4*) adult females at 24 hours post blood meal, as assessed by qRT-PCR. All values are the means ± SEM (n = 3). *$p < 0.05$, **$p < 0.01$, ***$p < 0.001$ from one-way ANOVA followed by Dunnett's multiple comparison test as compared to *iLuc* control.

Table S1. OATP proteins used for phylogenetic analysis.

Species	Protein name / Gene ID	GenBank accession number
Drosophila melanogaster	EcI/Oatp74D	NP_648989
	EcI-2/Oatp33Ea	NP_609568
	EcI-3/Oatp58Dc	NP_611659
	EcI-4/Oatp58Db	NP_611658
	Oatp26F	NP_609055
	Oatp30B	NP_723463
	Oatp33Eb	NP_609570
	Oatp58Da	NP_611657
Musca domestica	MDOA001191	XP_005183411
	MDOA012869	XP_011295428
	MDOA000381	XP_005176681
	MDOA000437	XP_005176680
	MDOA011793	XP_005176679
	MDOA005021	XP_011292793
	MDOA012671	XP_005184218
	MDOA001872	XP_005174920
Phlebotomus papatasi	PPAI001606	N.A.
	PPAI000868 (pseudogene)	N.A.
	PPAI000869	N.A.
	PPAI006402	N.A.
	PPAI006000	N.A.
	PPAI004257	N.A.
	PPAI005206 (pseudogene)	N.A.
	PPAI001455	N.A.
Aedes aegypti	EcI-2/AAEL007691	XP_001658583
	EcI-3/AAEL010921	XP_001661189
	EcI-4/AAEL010917	XP_001661188
	AAEL001812	XP_001660406
	AAEL001808	XP_001660407
	AAEL007693	XP_001658582
Anopheles gambiae	AGAP010042	XP_319187
	AGAP006638	XP_316669
	AGAP006637	XP_316668
	AGAP008712	XP_314819
	AGAP008711	XP_557860
	AGAP010043	XP_319188
Culex quinquefasciatus	CPIJ006275	XP_001847741
	CPIJ005186	XP_001847079
	CPIJ005183	XP_001847076
	CPIJ001972	XP_001843749
	CPIJ001971	XP_001843748
	CPIJ001970	XP_001843747
	CPIJ006274	XP_001847740

Table S2. Embryonic lethality in *Drosophila* ecdysone importer mutants.

Cross #	Genes Mutated				Expected Homozygous Larval Ratio (%)	Number of Larvae Analyzed	Homozygous Larval Number	Homozygous Larval Ratio (%)	Embryonic Hatchability (%)
	EcI	*EcI-2*	*EcI-3*	*EcI-4*					
1	-	+	+	+	33.33	217	72	33.18	99.5
2	+	-	+	+	50.00	184	91	49.46	98.9
3	+	+	-	+	50.00	182	93	51.10	102.2
4	+	+	+	-	50.00	178	93	52.25	104.5
5	+	-	-	+	33.33	228	73	32.02	96.1
6	+	-	+	-	33.33	117	43	36.75	110.3
7	+	+	-	-	50.00	176	88	50.00	100.0
8	+	-	-	-	33.33	139	46	33.09	99.3
9	-	-	+	+	16.67	409	75	18.34	110.0
10	-	+	-	+	16.67	286	47	16.43	98.6
11	-	+	+	-	16.67	154	28	18.18	109.1
12	-	-	-	+	11.11	290	30	10.34	93.1
13	-	-	+	-	11.11	124	13	10.48	94.4
14	-	+	-	-	16.67	193	33	17.10	102.6
15	-	-	-	-	11.11	122	13	10.66	95.9

Table S3. Post-embryonic lethality in *Drosophila* ecdysone importer mutants.

Cross #	Genes Mutated				Expected Homozygous Adult Ratio (%)	Number of Adults Analyzed	Homozygous Adult Number	Homozygous Adult Ratio (%)	Adult Viability (%)
	EcI	*EcI-2*	*EcI-3*	*EcI-4*					
1	-	+	+	+	33.33	-	0	0.00	0.0
2	+	-	+	+	50.00	365	186	50.96	101.9
3	+	+	-	+	50.00	269	131	48.70	97.4
4	+	+	+	-	50.00	353	174	49.29	98.6
5	+	-	-	+	33.33	161	56	34.78	104.3
6	+	-	+	-	33.33	163	55	33.74	101.2
7	+	+	-	-	50.00	340	179	52.65	105.3
8	+	-	-	-	33.33	185	64	34.59	103.8
9	-	-	+	+	16.67	-	0	0.00	0.00
10	-	+	-	+	16.67	-	0	0.00	0.00
11	-	+	+	-	16.67	-	0	0.00	0.00
12	-	-	-	+	11.11	-	0	0.00	0.00
13	-	-	+	-	11.11	-	0	0.00	0.00
14	-	+	-	-	16.67	-	0	0.00	0.00
15	-	-	-	-	11.11	-	0	0.00	0.00

Table S4. Oligonucleotides used for *Drosophila* study.

**Primers for qRT-PCR**			
Gene Name	CG Number	Forward (5'-3')	Reverse (5'-3')
Ecl-2	*CG5427*	ATCTACGGAGCTGGTCACGAGGTG	TTGTCCACTCCACAGAGTCGCTCG
Ecl-3	*CG3380*	AGAGCGAGAATCCCAGTAGCCTGG	TTCGGAGTGGTCTCTTCACCGTC
Ecl-4	*CG3382*	CTACGCTAGTCGAGGACATCGTCC	TGTCAGCCGCAAAGCTTCTTCGCC
rp49	*CG7939*	AGCTGTCGCACAAATGGCGCAAGC	TTGAATCCGGTGGGCAGCATGTGG
**Oligonucleotides for generating *Oatp CRISPR/Cas9* mutant target gRNAs**			
Allele	Target ID	Forward (5'-3')	Reverse (5'-3')
Ecl-2[1]	Target pair-I (T1)	CTTCGGGGTAGCTCACCATGCGGA	AAACTCCGCATGGTGAGCTACCCC
	Target pair-II (T2)	CTTCGGACATTTATGGCGACAAGG	AAACCCTTGTCGCCATAAATGTCC
Ecl-3[1]	Target pair-I (T1)	CTTCGTCAGTCAGTCAGTCCCGAA	AAACTTCGGGACTGACTGACTGAC
	Target pair-II (T2)	CTTCGCAACACATCACGGCCAAAA	AAACTTTTGGCCGTGATGTGTTGC
Ecl-4[1]	Target pair-I (T1)	CTTCGATCTCTTCACTGGCCATGA	AAACTCATGGCCAGTGAAGAGATC
	Target pair-II (T2)	CTTCGCTGCCCAGGAAAATCAGAG	AAACCTCTGATTTTCCTGGGCAGC
Ecl-3-4[1]	Target pair-I (T1)	CTTCGTCAGTCAGTCAGTCCCGAA	AAACTTCGGGACTGACTGACTGAC
	Target pair-II (T2)	CTTCGCTGCCCAGGAAAATCAGAG	AAACCTCTGATTTTCCTGGGCAGC
**Primers for screening *CRISPR/Cas9* mutants**			
Allele		Forward (5'-3')	Reverse (5'-3')
Ecl-2[1]		TAGTTAGCAGCACTTCACTCGC	TTGAGTATCACGGATGGGTTGC
Ecl-3[1]		AAGTTCAAGGGGCGAAGTTGTG	ATGGCAATAAAGACAGCTGCGG
Ecl-4[1]		GACAATTAACGACCAGATCCCG	TGGAAACGTGCGAAATTCTCGC
Ecl-3-4[1]		AAGTTCAAGGGGCGAAGTTGTG	TGGAAACGTGCGAAATTCTCGC
**Primers for pcDNA3.1(+) cloning**			
Gene ID		Forward (5'-3')	Reverse (5'-3')
Ecl-4		TGTAGATCTGCCACCATGGCCAGTGAAGAG ATCG	CGGACTCGAGTTATTTCTTTTTAGTAACAA CATC

Table S5. Oligonucleotides used for *Aedes* study.

**Primers for qRT-PCR**		
Gene ID	Forward (5'-3')	Reverse (5'-3')
EcI-2/AAEL007691	AGGACACAGAAATCGTCCCCGGTG	TCCCGCTCCGTAGATGAAGTGTGG
EcI-3/AAEL010921	ATCTGTTGTACGGTCCGGGAGAGG	TCCTCGAGTTCCATTACCCCGGCA
EcI-4/AAEL010917	ACAGTGAACGTGACGTGCAGTGCG	ATCCGACCCTGCCGTGATGATTCC
AAEL001812	ACTACACGATGGCCGTCGTTGGTC	TGCTGTGTGGCGTTAGTCCCAAGG
AAEL001808	ACACCGAACCAGAGGAATCGCTGC	TCCTGGCGAACTTCTGAATCGCCG
AAEL007693	ACATTGGATGTGCCCTGGGTGCTC	ATGATGTCATCTGCCGCTTGCCGG
RpL32/AAEL003396	AAGTTCCTGGTCCACAACGTGCGC	TGCTTGCGCTTCTTGGACGACACG
Luciferase	AGCACTCTGATTGACAAATACGA	AGTTCACCGGCGTCATCGTC
Vg/AAEL010434	GCAGGAATGTGTCAAGCGTGA	ACGAGGACGAAGAATCGGAAG
E74B/AAEL000741	GCCACTCAGGAATATCTGGAAC	GGAAAGTGGGACGATGGATAAC
**Primers for dsRNA synthesis. T7 promoter sequence (5'-TAATACGACTCACTATAGGGAGA-3') was added to the 5' end of each primer.**		
Gene ID	Forward (5'-3')	Reverse (5'-3')
EcR/AAEL019431	AAGCGAGGTTATGATGTTGCGAATG	TGAGGACGAGGACTGGGTGCC
EcI-2/AAEL007691	GGCTCGGGTGGATCATACT	AAGAATCTTCGGCGAGGG
EcI-3/AAEL010921	CATTCGCGGAAAGCTGTTATT	GTAACTGAGGATGGCTGATAGG
EcI-4/AAEL010917	CGTGTTCTTATTCGGTATAG	ACGTATGCTTTGTTAGTAAT
Shade/AAEL010946	ATCTCCACAAACGTTACGGC	GTTCTTTTGCTTGTCTCCGC
**Primers for checking RNAi efficiency**		
Gene ID	Forward (5'-3')	Reverse (5'-3')
EcR/AAEL019431	AAGCGAGGTTATGATGTTGCG	CAGCAGGTCCTCTATCGTGTCC
EcI-2/AAEL007691	CTCAGGCATTGTCATCTC	GGGCACGTTAGGAATAAG
EcI-3/AAEL010921	ACGATTACAACCCTAGAA	ACCGAATGCTATCCATC
EcI-4/AAEL010917	GCTGGATTTCCAAAGATT	CTCGCCGTATTATTGAAC
Shade/AAEL010946	TCCAGGACATTGCGAACAC	AGGTCGATTTCACGGCTTC
**Oligonucleotides for generating *CRISPR/Cas9* mutant target gRNAs**		
Oligo ID	Forward (5'-3')	
EcR/AAEL019431-F	GAAATTAATACGACTCACTATAGGCGAGATGGACATGTACATGGTTTTAGAGCTAGAAATAGC	
EcI-2/AAEL007691-F	GAAATTAATACGACTCACTATAGGCCAACAAGATAATCCACAAGTTTTAGAGCTAGAAATAGC	
EcI-3/AAEL010921-F	GAAATTAATACGACTCACTATAGGATAACGATGACGATGTGGGGTTTTAGAGCTAGAAATAGC	
EcI-4/AAEL010917-F	GAAATTAATACGACTCACTATAGGTGTAGGAAATGTTACGACGGTTTTAGAGCTAGAAATAGC	
sgRNA-R	AAAAGCACCGACTCGGTGCCACTTTTTCAAGTTGATAACGGACTAGCCTTATTTTAACTTGCTATTTCTAGCTCTAAAAC	
**Primers for screening *CRISPR/Cas9* mutants**		
Allele	Forward (5'-3')	Reverse (5'-3')
EcR/AAEL019431	TGAGGATCTATCGCCTTCCA	AGGTGCTGTTCGTTGTACTG
EcI-2/AAEL007691	GTTGTCGAAAGGTGTCAG	TATTCCAGCCTCTGATCG
EcI-3/AAEL010921	CTTCGTCAGTGGCATGTCCT	AGACCCGCAAGAATTGTCGT
EcI-4/AAEL010917	GTCCGATGACCCATAGCCTG	TTTGCCGCACTTTCTGTTCG
**Primers for pcDNA3.1(+) cloning**		
Gene ID	Forward (5'-3')	Reverse (5'-3')
EcI-2/AAEL007691	TGTGGATCCGCCACCATGAAATCCAACTACCTTTG	TTAGAATTCTCACTTGAATGCGCTTTTGGATTG
EcI-3/AAEL010921	ACAGCTAGCGCCACCATGGGTCTGGAAAACAAG	CTTGGATCCCTATGACGGTCGATCAGTTGTG
EcI-4/AAEL010917	TGTGGATCCGCCACCATGTTTGGCAGTGGAATAC	CTACTCGAGTTATTCACGCTCATTTTGTTCC
AAEL001812	TGTGGATCCGCCACCATGTCGTCAACAAGTTTGG	TTAGAATTCTCAATATTTGTCCACGTGGTGCGC
AAEL001808	GCAAAGCTTGCCACCATGAAGATGACAACCTCC	CTTGGATCCCTAAACATCCGTTGACTTCGAGC
AAEL007693	ACTAAGCTTGCCACCATGGGCGACTACAAAGAAA	TTAGAATTCCTAAAAGTCCGTTTCCGGACTCATC
